# SPHK1 promotes bladder cancer metastasis via PD-L2/c-Src/FAK signaling cascade

**DOI:** 10.1038/s41419-024-07044-3

**Published:** 2024-09-16

**Authors:** Wei-Hsiang Kao, Li-Zhu Liao, Yu-An Chen, U-Ging Lo, Rey-Chen Pong, Elizabeth Hernandez, Mei-Chih Chen, Chieh-Lin Jerry Teng, Hsin-Yi Wang, Stella Chin-Shaw Tsai, Payal Kapur, Chih-Ho Lai, Jer-Tsong Hsieh, Ho Lin

**Affiliations:** 1 Department of Life Sciences, National Chung Hsing University, Taichung, Taiwan; 2 Department of Urology, University of Texas Southwestern Medical Center, Dallas, TX USA; 3 Cancer Genome Research Center, Chang Gung Memorial Hospital at Linkou, Taoyuan, Taiwan; 4 Translational Cell Therapy Center, China Medical University Hospital, Taichung, Taiwan; 5 Division of Hematology/Medical Oncology, Department of Internal Medicine, Taichung Veterans General Hospital, Taichung, Taiwan; 6 Department of Post-Baccalaureate Medicine, College of Medicine, National Chung Hsing University, Taichung, Taiwan; 7 Department of Nuclear Medicine, Taichung Veterans General Hospital, Taichung, Taiwan; 8 Superintendent Office, Tungs’ Taichung MetroHarbor Hospital, Taichung, Taiwan; 9 College of Life Sciences, National Chung Hsing University, Taichung, Taiwan; 10 Urology and Pathology, University of Texas Southwestern Medical Center, Dallas, TX USA; 11 Department of Microbiology and Immunology, Chang Gung University, Taoyuan, Taiwan; 12 Rong Hsing Research Center for Translational Medicine, National Chung Hsing University, Taichung, Taiwan

**Keywords:** Bladder cancer, Metastasis, Lipid signalling, Targeted therapies

## Abstract

SPHK1 (sphingosine kinase type 1) is characterized as a rate-limiting enzyme in sphingolipid metabolism to phosphorylate sphingosine into sphingosine-1-phosphate (S1P) that can bind to S1P receptors (S1PRs) to initiate several signal transductions leading to cell proliferation and survival of normal cell. Many studies have indicated that SPHK1 is involved in several types of cancer development, however, a little is known in bladder cancer. The TCGA database analysis was utilized for analyzing the clinical relevance of SPHK1 in bladder cancer. Through CRISPR/Cas9 knockout (KO) and constitutive activation (CA) strategies on SPHK1 in the bladder cancer cells, we demonstrated the potential downstream target could be programmed cell death 1 ligand 2 (PD-L2). On the other hand, we demonstrated that FDA-approved SPHK1 inhibitor Gilenya® (FTY720) can successfully suppress bladder cancer metastasis by in vitro and in vivo approaches. This finding indicated that SPHK1 as a potent therapeutic target for metastatic bladder cancer by dissecting the mechanism of action, SPHK1/S1P-elicited Akt/β-catenin activation promoted the induction of PD-L2 that is a downstream effector in facilitating bladder cancer invasion and migration. Notably, PD-L2 interacted with c-Src that further activates FAK. Here, we unveil the clinical relevance of SPHK1 in bladder cancer progression and the driver role in bladder cancer metastasis. Moreover, we demonstrated the inhibitory effect of FDA-approved SPHK1 inhibitor FTY720 on bladder cancer metastasis from both in vitro and in vivo models.

## Introduction

Bladder cancer is the seventh most common cancer in the USA, and it ranks as the ninth cancer type worldwide [1]. Possible risk factors are smoking, advanced age, environmental carcinogen exposure, gene mutation, *Schistosoma haematobium* infection, and others [2–5]. Almost 90% of bladder cancer is transitional cell carcinoma (TCC); the rest includes squamous cell carcinoma, adenocarcinoma, small cell carcinoma, and sarcoma. In general, 75% of patients are non-muscle-invasive bladder cancer (NMIBC), and 25% of patients are muscle-invasive bladder cancer (MIBC) [6]. Despite the high recurrence of NMIBC (50–70%), the probability of invasive progression is around 10–15%, and the 5-year survival rate is about 90% [7]. In contrast to NMIBC, the 5-year survival rate of MIBC patients is significantly low (60–70%) [8]. The most common distant metastasis sites of bladder cancer are lymph nodes, bone, urinary, lung, liver, and retroperitoneum, which account for 50% of patients with MIBC [9, 10]. Noticeably, the median survival rate is around 3~6 months in untreated patients with distant metastases [11]. About 30–70% of NMIBC patients show a risk of recurrence, and up to 30% of patients progress to muscle-invasive disease within 5 years. Although the radical cystectomy plus lymph node dissection would be applied on localized MIBC patients after neoadjuvant chemotherapy, the long-term survival rate remains unchanged [12]. Due to the significant cisplatin-induced nephrotoxicity, it leads to about half of the MIBC patients failed to match eligibility criteria for cisplatin-related therapy [13–16]. The recently approved Enfortumab Vedotin (EV), an antibody–drug conjugate, in combination with Pembrolizumab for the treatment of cisplatin-ineligible patients with locally advanced or metastatic bladder cancer, but overall response rate is still unknown [17, 18]. In addition, Erdafitinib (BALVERSA™) was approved and served as second-line treatment for patients with locally advanced or metastatic bladder cancer with FGFR mutation, it has been found that only around 14% of patients’ FGFR3 is frequently mutated, and 2% of tumors have gene fusion [19]. Hence, identifying new drivers of bladder metastasis is urgent and critical for potential cancer treatment in the future.

It has been found that altered lipid metabolism is among the most prominent alteration in many different types of cancer, including endometrial, colorectal, gastric, liver, prostate, and bladder cancer [20]. In addition to classical lipogenesis, sphingolipids are an extensive class of lipids with different functions in the cell such as ceramides are usually mediating antiproliferative responses, through inhibiting cancer cell growth and migration, as well as inducing autophagy and apoptosis [20]. In contrast, sphingosine-1-phosphate (S1P) produced by sphingosine kinase (SPHK) plays the opposite role by activating the specific S1P receptors (S1PRs), which induces cancer cell transformation, migration and growth and promotes cancer evasiveness and drug resistance. Although clinical prevalence of SPHK1 expression in bladder cancer is highly significant [21], the detailed biologic actions and molecular mechanisms of SPHK1 in bladder cancer development are not fully characterized.

In this study, we demonstrated the driver role of SPHK1 in promoting in vitro migration and invasion as well as lung metastases of bladder cancer in which S1PR-elicited Akt/β-catenin signaling network is the key downstream effector for the mechanism of action. Through mining TCGA database and performed gene set enrichment analysis (GSEA), we identified PD-L2 induced by S1P can interact with c-Src and FAK underlying bladder cancer migration and invasion. Moreover, we further examined the efficacy of FDA-approved SPHK1 inhibitor (i.e., FTY720) in reducing bladder cancer metastasis using in vivo orthotopically metastatic xenograft model. These findings not only unveil a new functional role of PD-L2 regulated by S1PR/Akt/β-catenin signaling network in facilitating bladder cancer metastases, but also identify SPHK1 as a potential therapeutic target for preventing bladder cancer progression.

## Materials and methods

### Cell culture and reagents

Human TCC cell lines 253J and UMUC13 were purchased from ATCC (Manassas, VA). The T24L, a T24 lung metastatic subline, was generated as previously described [22]. The 253J-BV, 253J metastatic subline, was obtained from Dr. Colin Danney, MD Anderson Cancer Center [23]. Cells were maintained in RPMI-1640 medium (Sigma-Aldrich, MO) supplemented with 10% fetal bovine serum and 1% penicillin/streptomycin and incubated at 37 °C with 5% CO_2_. All these cell lines were authenticated with the short tandem repeat profiling by Genomic Core in UT Southwestern (UTSW) periodically, and mycoplasma testing was performed by MycoAlert® kit (Lonza Walkersville, Inc. Walkersville, MD) every quarterly to ensure mycoplasma-free. S1P was purchased from TOCRIS (Minneapolis, MN). SPHK1 inhibitor (FTY720) and FAK inhibitor (VS-6063) were purchased from Selleckchem (Houston, TX). PI3K/Akt inhibitor (LY294002), ERK inhibitor (PD98059), and c-Src inhibitor (Dasatinib) were purchased from Sigma-Aldrich (MO). NF-кB inhibitor (BAY 11-7082) was purchased from Santa Cruz (Dallas, TX).

### PD-L2 expression construct establishment

The cDNA template is generated from total RNA extracts of T24L cells by RT-PCR. The primers with XbaI and KpnI cleavage sites were used for PD-L2 amplification: hPDCD1LG2-F: 5′-GGTACCAACATGATCTTCCTCCTGCTAATGT-3′; hPDCD1LG2-R: 5′-CACTCTAGATCAGATAGCACTGTTCACTTCCC-3′. Samples were amplified by 25 cycles, and the amplified fragments were digested by XbaI and KpnI restriction enzymes and ligated into the pcDNA3.1 expression vector.

### CRISPR/Cas9 gene knockout, lentivirus production, and gene transfection

The sgRNA sequence for the SPHK1 gene was chosen from the CHOPCHOP web tool (https://chopchop.cbu.uib.no/): the target sequence (5′-GCGGGTTCAGCAGCACCAGCACG-3′) is in the exon 3 of SPHK1 gene. The sgRNA oligos were designed (oligo 1: 5′-CACCGGCGGGTTCAGCAGCACCAGCACG-3′ and oligo 2: 5′-AAACCGTGCTGGTGCTGCTGAACCCGCC-3′) to establish the letiCRISPRv2-sgSPHK1-exon3 construct. Briefly, the lentiCRISPRv2 plasmid was digested by Esp3I (NEB, Ipswich, MA) at 37 °C for 30 min. Meanwhile, the oligos were phosphorylated and annealed by T4 polynucleotide kinase (NEB, Ipswich, MA) at 37 °C for 30 min. Both lentiCRISPRv2 and oligos were incubated with T4 ligase at 22 °C for 2 h and then transformed into stable *E. coli* competent cells (NEB, Ipswich, MA) for 20–24 h at 30 °C. Plasmid from each colony was extracted using GeneJET Plasmid Miniprep Kit (Invitrogen, Waltham, MA) and subjected to DNA sequencing for validation. The lentivirus was harvested from the culture media of HEK293t cells 48 h after co-transfecting with LentiCRISPRv2-sgSPHK1-exon3, psPAX2 [encoding *gag* (group-specific antigen), *pol* (viral reverse transcriptase), *rev* (regulator of expression of virion proteins), and *tat* (HIV trans-activator)], and pMD2.G (encoding VSV-G envelope protein). LentiCRISPRv2-sgNT plasmid was used to control lentivirus production. For knockout of PD-L2, the target sequences (5′-CCACATACCTCAAGTCCAAGTGA-3′; 5′-CACACAGCTGAAGTTTCTGCCAG-3′; 5′-TTGCCCAGGTCAGATGGAACCC-3′; 5′-AAAAGACCTGTCACCACAACAAAG-3′) are located in exon 3, 4, 5, and 6. The sgRNA oligos were designed (oligo 3-F: 5′-CACCGCCACATACCTCAAGTCCAAGTGA-3′ and oligo 3-R: 5′- AAACTCACTTGGACTTGAGGTATGTGGC-3′; oligo 4-F: 5′-CACCGCACACAGCTGAAGTTTCTGCCAG-3′ and oligo 4-R: 5′-AAACCTGGCAGAAACTTCAGCTGTGTGC-3′; oligo 5-F: 5′- CACCGTTGCCCAGGTCAGATGGAACCC-3′ and oligo 5-R: 5′-AAACGGGTTCCATCTGACCTGGGCAAC-3′; oligo 6-F: 5′- CACCGAAAAGACCTGTCACCACAACAAAG-3′ and oligo 6-R: 5′-AAACCTTTGTTGTGGTGACAGGTCTTTTC-3′) in order to establish the letiCRISPRv2-sgPD-L2-exon3, letiCRISPRv2-sgPD-L2-exon4, letiCRISPRv2-sgPD-L2-exon5, and letiCRISPRv2-sgPD-L2-exon6 constructs. SPHK1-knockout T24L (T24LsgSPHK1), 253J-BV (253J-BVsgSPHK1), and PD-L2-knockout 253J-BV (253J-BVsgPD-L2) sublines were generated by incubating lentivirus for 48 h then selected with Puromycin for at least 2 weeks to select the stable clones. Gene transfection with either pcDNA3.1-neo, pcDNA3.1-SPHK1-S225E (CA) plasmid [24], or pcDNA3.1-PD-L2 was carried out using Xfect transfection regent (TaKaRa, CA) according to the manufacturer’s protocol, and G418 was used for stable clone selection.

### Quantitative real-time PCR (qRT-PCR)

Total RNA was extracted by Maxwell 16 LEV SimplyRNA Purification Kit (Promega, Madison, WI), and 2 μg RNA was reversely transcribed into cDNA by iScript cDNA Synthesis Kit (Bio-Rad, Hercules, CA). PCR analysis was carried out in CFX384 Touch Real-Time PCR System (Bio-Rad, Hercules, CA) using iTaq Universal SYBR Green Supermix (Bio-Rad, Hercules, CA). The relative mRNA level of each gene was determined by normalizing with 18S rRNA. The qRT-PCR primer sequences for targets are as follows: 18S rRNA (F: 5′-GGCGGCGTTATTCCCATGA-3′, R: 5′-GAGGTTTCCCGTGTTGAG-3′), SPHK1 (F: 5′-TTCCTTGAACCATTATGCTG-3′, R: 5′-GATACTTCTCACTCTCTAGGTC-3′), SPHK2 (F: 5′-ATGGCATCGTCACGGTCTC-3′, R: 5′-CTCCCAGTCAGGGCGATCTA-3′), PD-L1 (F: 5′-AAACAATTAGACCTGGCTG-3′, R: 5′- TCTTACCACTCAGGACTTG-3′), PD-L2 (F: 5′-ATTGCAGCTTCACCAGATAGC-3′, R: 5′-AAAGTTGCATTCCAGGGTCAC-3′), FAK (F: 5′- TGGTGCAATGGAGCGAGTATT-3′, R: 5′-CAGTGAACCTCCTCTGACCG-3′).

### Western blot analysis

Cells were lysed in RIPA buffer [150 mM sodium chloride, 1% Triton X-100, 1% sodium deoxycholate, 0.1% SDS, 50 mM Tris-HCl (pH 7.5), 2 mM EDTA (pH 8.0), protease inhibitor cocktail (Thermo Fisher Scientific, MA)]. Total protein lysates were obtained by centrifugation at 4 °C for 20 min at 15,400 × *g*. An equal amount of protein was loaded into Bolt™ Bis-Tris Plus Gels (Thermo Fisher Scientific, MA) for protein separation and then transferred onto nitrocellulose membranes by Trans-Blot Turbo Transfer System (Bio-Rad, Hercules, CA). Membranes were incubated with 2% BSA (w/v) for an hour and then incubated with primary antibodies overnight. After treatment with the secondary antibodies conjugated with horseradish peroxidase, enhanced chemiluminescence (Advansta, San Jose, CA) was used for detecting the signal. The antibodies for Actin (sc-58673), Tubulin (sc-32293), GAPDH (sc-166574), β-catenin (sc-7963), c-Src (sc-8056), and FAK (sc-558) were purchased from Santa Cruz (Dallas, TX); SPHK1 (#12071), pS473-Akt (#4060), Akt (#4691), PD-L1 (#13684) were purchased from Cell Signaling (Danvers, MA); SPHK2 (#PA5-51064) and Y419-Src (#44-660 G) were purchased from Invitrogen (Waltham, MA); PD-L2 (ab187662) was purchased from Abcam (Waltham, MA); Y397-FAK (611723) was purchased from BD Biosciences (Milpitas, CA). Secondary goat anti-mouse (115-035-003) or goat anti-rabbit (111-035-003) antibodies were purchased from Jackson ImmunoResearch (West Grove, PA).

### Cell growth assay

Two thousand cells were seeded into a 96-well plate with 200 μl medium and incubated at 37 °C for 24 h. Ten percent medium volume of MTT reagent (5 mg/ml in PBS, Sigma-Aldrich, MO) was added into each well for 3 h of incubation then DMSO (Sigma-Aldrich, MO) was used to lyse the cells. The absorbance of samples was read spectrophotometrically at OD 570 nm by Epoch Microplate Spectrophotometer (BioTek, Winooski, VT), and cell viability was analyzed.

### Migration and invasion assay

For migration assay, 20,000–40,000 cells were plated onto the upper chamber of Transwell (8 µm pore size, Corning, NY) with 300 μl medium, and 1 ml medium was added into the lower chamber for 24 h incubation. For invasion assay, 100 μl 2% Matrigel/serum-free medium was coated in the Transwell chamber for 4 h at 37 °C and then performed a similar procedure as above. Cells were fixed with 4% paraformaldehyde for 10 min and stained with 0.05% Crystal violet solution for 30 min. The chambers were washed with ddH_2_O and dried overnight. Data were analyzed by the Keyence microscope (BZ-X700, Itasca, IL).

### Animal models and experimental therapy

Mice were randomly divided into each group. Two T24L sublines (T24LsgNT and T24LsgSPHK1; 1 × 10^6^ cells) were intravenously injected into 6-week-old female SCID mice to compare their metastatic potential to the lung. Bioluminescence imaging (BLI) was carried out weekly to monitor tumor growth using IVIS Spectrum in vivo imaging system (Caliper Life Sciences, Hopkinton, MA). Mice were sacrificed 3 weeks after injection then lung tissues were excised for ex vivo BLI imaging and preserved in 10% formalin-buffered saline for H&E staining. Tumor nodules presented in the lung parenchyma were observed by Keyence microscopy, and the number and size of nodules were counted and analyzed by ImageJ software. To generate orthotopic bladder cancer model, 1 × 10^6^ T24L cells were instilled into the bladder of 6-week-old female SCID mice using BD Angiocath™ (24G ×0.75 in, BD, Franklin Lakes, NJ) and lung metastasis was monitored by IVIS Spectrum in vivo imaging system. After 2 weeks of cell inoculation, FTY720 (5 mg/kg) was administered by intraperitoneal injection (IP) three times per week for 4 weeks. Both primary tumor and lung metastatic nodules were monitored by IVIS Spectrum in vivo imaging system weekly then mice were sacrificed after four cycles of treatment. All animal work was approved by the Institutional Animal Care and Use Committee.

### Immunoprecipitation

Cell lysates were harvested by lysis buffer and then the protein of interest was isolated by a specific antibody conjugated to Protein A Mag Sepharose™ Xtra (Cytiva, Washington, DC). Briefly, 500 µg of lysates from each sample was subjected to a specific antibody with Protein A Mag Sepharose™ Xtra in a ratio of 1 µg: 10 µl for overnight rotation. After three times of PBS wash, immunoprecipitated proteins were analyzed by western blotting.

### Bioinformatics data analyses

For analyzing the clinical correlation of SPHK1 mRNA expression between cancer tissues (*N* = 408) and normal tissues (*N* = 19), tumor stage, and subtypes, RNA-seq data derived from the TCGA database were obtained from the Firebrowse website (http://firebrowse.org/). The figures and statistics results (Normal vs Cancer: *p* = 0.000002479; Benign vs Cancer: *p* = 0.03504) were carried out by Prism 9.0 (GraphPad, San Diego, CA). The overall survival rate (*p* = 0.00017) was generated from the TCGA database GEPIA2 website (http://gepia2.cancer-pku.cn/). The histologic grade (*p* = 0.00124) was analyzed from cBioPortal, which is based on the database: whole-exome sequencing of 131 high-grade muscle-invasive urothelial bladder carcinomas (2014) [25].

### Statistical analyses

All data represent mean ± standard deviation (SD). Each *n* value is described in the corresponding figure legend. GraphPad Prism software was used for determining the statistical relevance between groups by one-tailed Student’s *t*-test, statistical significance from three or more groups was calculated by one-way ANOVA. Asterisks represent statistical significance (*p < 0.05; **p < 0.01; ***p < 0.001; ****p < 0.0001).

## Results

### Clinical correlation between SPHK1 and bladder cancer development

**Fig. 1:**
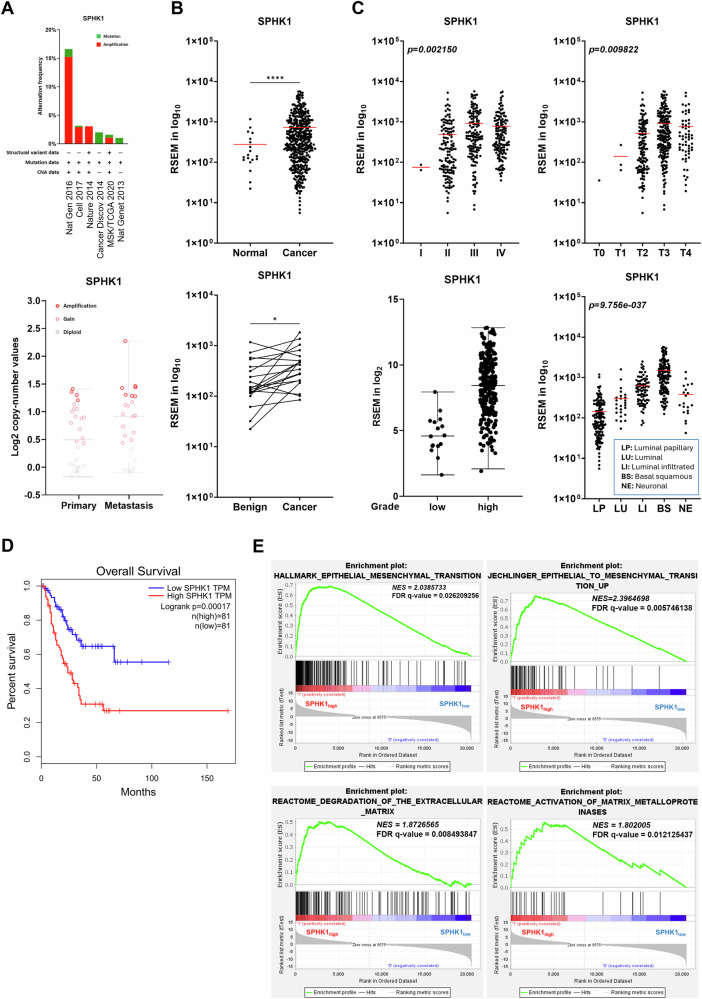
Clinical correlation between SPHK1 and bladder cancer development. **A** The frequency of SPHK1 gene alternation and the status of SPHK1 gene copy number from primary and metastatic samples analyzed from six different bladder cancer TCGA dataset in cBioPortal. **B** SPHK1 expression in normal bladder vs cancer samples from TCGA database (Benign = 19, Tumor = 408). *p < 0.05, ****p < 0.0001. **C** The expression of SPHK1 in bladder cancer samples from the TCGA database based on stage, T category in TNM classification, grade, and different subtypes. One-way ANOVA was performed (*p < 0.05, ****p < 0.0001). LP luminal papillary, LU luminal, LI luminal infiltrated, BS basal squamous, NE neuronal. **D** Significantly different overall survival of bladder cancer patients with high and low SPHK1 expression based on GEPIA website (***p < 0.001). **E** GSEA analysis of migration/invasion-related pathway in bladder cancer samples from the TCGA database (high SPHK1 group = 117 and low SPHK1 group = 282).

S1P signaling is frequently linked to malignancies of tumor cells. Here, by analyzing bladder cancer TCGA database, although small percentage of SPHK1 gene amplification was detected (Fig. 1A), a significantly elevated SPHK1 expression in cancer tissues compared with normal tissues as well as paired samples (Fig. 1B). Also, there is a positive correlation between SPHK1 expression and tumor-grade or -stage (Fig. 1C). Noticeably, elevated SPHK1 is associated with the aggressive-type basal squamous (BS) than other non-aggressive-type luminal type of sublines such as luminal papillary (LP), luminal (LU), and luminal infiltrated (LI) (Fig. 1C). Moreover, patients with high SPHK1 expression have a poor overall survival than those with low SPHK1 expression (Fig. 1D). Based on GSEA of RNA-seq data from 399 patients, the data indicated that the sets of epithelial–mesenchymal transition (EMT) genes and extracellular matrix modulation-related signaling were enriched in the high SPHK1 group (Fig. 1E). On the other hand, SPHK2, another isoform of SPHK, did not exhibit any positive correlation in terms of tumor subtypes, grade, stage, disease progression, and overall survival rate (Fig. S1). Taken together, SPHK1 but not SPHK2 is likely involved in bladder oncogenesis, particularly, in disease progression.

### The regulation of in vitro cell migration and invasion by SPHK1

**Fig. 2:**
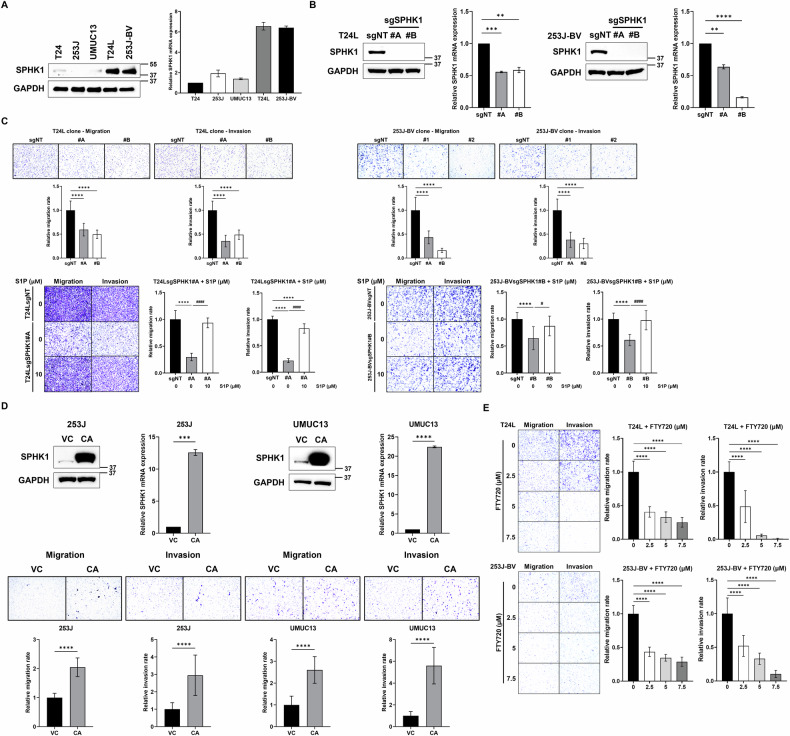
The regulation of cell migration and invasion by SPHK1. **A** The profile of SPHK1 mRNA and protein expression in 253J-BV bladder cancer cell lines (*n* = 3). **B** The characterization of SPHK1 mRNA and protein expression in SPHK1-KO sublines of T24L and 253J-BV cell lines (*n* = 3). **C** The effect of SPHK1 on cell migration and invasion of different bladder cancer cell models (*n* = 3). **D** The effect of constitutive activate (CA) SPHK1 on cell migration and invasion of 253J and UMUC13 cell lines (*n* = 3). **E** The inhibitory effect of SPHK1 inhibitor (FTY720) on cell migration and invasion of T24L and 253J-BV cell lines (*n* = 3). All results were presented as mean ± SD and statistically calculated (^#^*p* < 0.05, **p < 0.01, ***p < 0.001, ^####^*p* < 0.0001, ****p < 0.0001).

As shown in Fig. 2A, by profiling SPHK1 protein expression among different bladder cancer cell lines (i.e., T24, 253J, UMUC13, T24L [T24 lung metastatic subline], and 253J-BV [253J lung metastatic subline]), the data indicated that highly elevated SPHK1 was detected in T24L and 253J-BV compared with their parental cells. Thus, the SPHK1 gene knockout (KO) clones from both sublines were established using CRISPR/Cas9 technology (Fig. 2B) then subjected to Transwell well for determining cell migratory and invasion ability. The data demonstrated that significantly decreased cell migration and invasion in SPHK1 KO cells (Fig. 2C). Noticeably, by adding S1P exogenously could restore cell migratory and invasive abilities (Fig. 2C). In parallel, 253J and UMUC13 cells were transfected with SPHK1 constitutively activating (CA) construct (i.e., S225E) (Fig. 2D), and the results clearly indicated significantly increased cell migration and invasion of SPHK1-CA cells (Fig. 2D). Furthermore, T24L and 253J-BV cells were treated with specific inhibitor of SPHK1 (i.e., FTY720) and a dose-dependent inhibitory effect of FTY720 on both cell migration and invasion of T24L and 253J-BV cells (Fig. 2E); these concentrations were not toxic to these cells (Fig. S2). These in vitro data support the potential impact of SPHK1 elevation on bladder cancer progression.

### The impact of SPHK1 on in vivo lung metastasis of bladder cancer

**Fig. 3:**
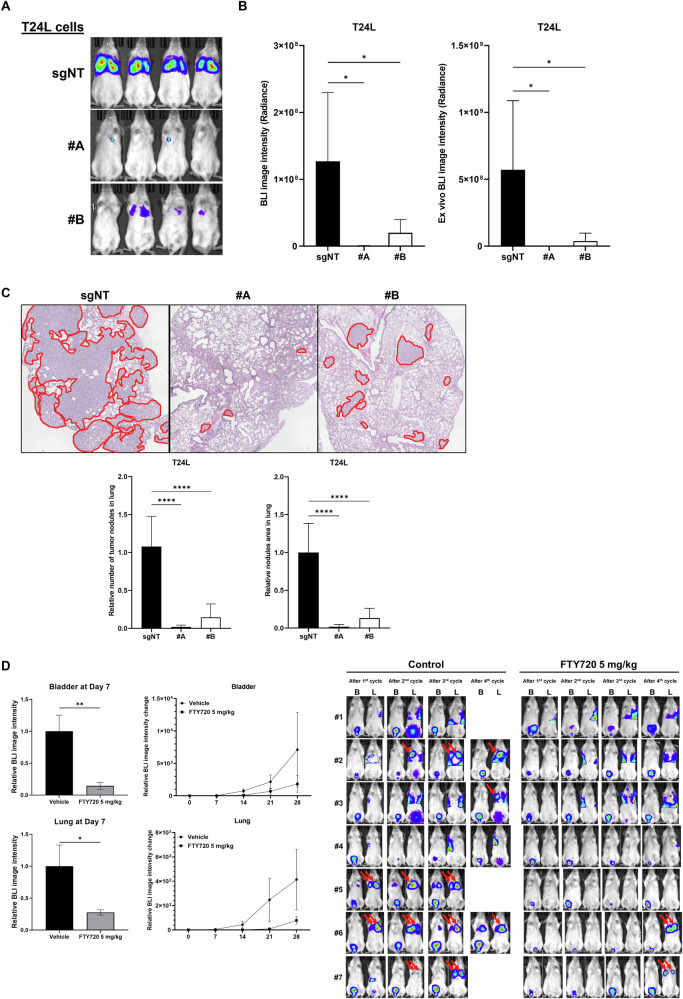
The effect of FTY720 on SPHK1-promoting lung metastases of bladder cancer. **A** Determination of lung metastases of T24LsgNT, T24LsgSPHK1#A, and T24LsgSPHK1#B (1 × 10^6^ cells) injected intravenously into female SCID mice via tail vein by whole body BLI (IVIS Spectrum) (*n* = 4). **B** The relative imaging intensity of whole body BLI (left panel) and ex vivo BLI (right panel) (*n* = 4). **C** The pathological examination and determination of tumor nodules (circled in red) in lung metastases of T24L and SPHK1 KO sublines (*n* = 4). **D** The therapeutic effect of FTY720 on primary tumor and lung metastases of orthotopic model of T24L cells. After 2 weeks of cell implantation, IP administration of 5 mg/kg FTY720 (three times per week) was carried out for 2 weeks and followed by and weekly BLI (B: BLI signal from the bladder; L: BLI signal from the lung; red arrow: BLI signal from the lung metastatic nodule) (*n* = 7). The data were presented as mean ± SD and statistically calculated (*p < 0.05, **p < 0.01, ****p < 0.0001).

To examine the in vivo effect of SPHK1 on bladder cancer metastasis, xenograft models with lung metastatic sublines of T24L and SPHK1 KO (clones #A and #B) cells were conducted using intravenous injection or intravesical implantation. As shown in Fig. 3A, BLI data indicated that the significant reduction of light signal in the upper body part of animals injected with SPHK1 KO sublines compared with T24L sgNT cells, which was consistent with ex vivo imaging of lung tissues dissected from both SPHK1 KO sublines (Fig. 3B). The pathologic examination of lung tissues section with the H&E stain also showed both the number as well as the size of tumor nodules in the lung were significantly decreased in both SPHK1 KO sublines compared to sgNT subline (Fig. 3C). In addition, by applying pharmacologic intervention on an orthotopic bladder cancer model, the IP administration of FTY720 was able to reduce the incidence of lung metastases as well as the growth of primary tumor during the four-cycle treatment (Fig. 3D). Taken together, SPHK1 plays a potent driver role in bladder cancer progression.

### The role of PD-L2 in SPHK1-elicited cancer migration and invasion

**Fig. 4:**
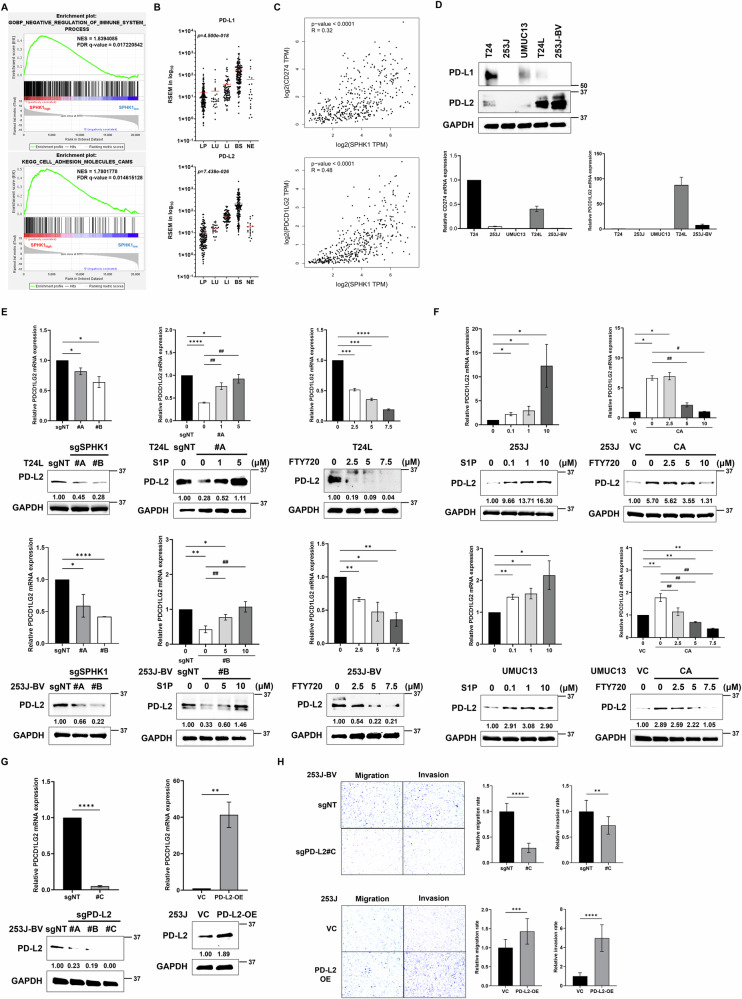
The role of PD-L2 in SPHK1-elicited cell migration and invasion of bladder cancer. **A** GSEA analysis of immune regulation and adhesion-related pathway using bladder cancer samples from TCGA database (high SPHK1 group = 117; low SPHK1 group = 282). **B** The profile of CD274 (PD-L1) or PDCD1LG2 (PD-L2) expression in different subtypes of bladder cancer from the TCGA database analyzed by one-way ANOVA. **C** The correlation of PD-L1 or PD-L2 with SPHK1 gene expression in bladder cancer samples from TCGA database. **D** The profile of PD-L1 and PD-L2 mRNA and protein expression in 253J-BV bladder cancer cell lines (*n* = 3). **E**, **F** The regulation of PD-L2 mRNA and protein expression by SPHK1 activities in bladder cancer cell lines cells (*n* = 3). **G**, **H** The effect of PD-L2 on cell migration and invasion of 253J-BV PD-L2 KO cells and 253J PD-L2 OE (*n* = 3). All results were presented as mean ± SD and statistically calculated (*p < 0.05, ^#^*p* < 0.05, **p < 0.01, ^##^*p* < 0.01, ***p < 0.001, ****p < 0.0001).

Through GSEA analysis, we find that the sets of genes about immune system process regulation is enriched in high SPHK1 group, and then we noticed that PD-L1 and PD-L2 were presented in the set of cell adhesion molecule genes enriched in high expression of SPHK1 group as well (Fig. 4A). From bladder cancer subtype analyses, we also found that PD-L1 and PD-L2 expressions were significantly elevated in BS group compared to the other groups such as LP, LU, and LI (Fig. 4B). In addition, there were positive correlation between PD-L1 or PD-L2 expression and SPHK1 expression by Pearson’s analyses from TCGA database (Fig. 4C). Based on these bioinformatics analyses, we found that PD-L2 expression was high in two metastatic T24L and 253J-BV cells and 253J and UMUC13 had low PD-L2 expression, and parental T24 had intermediate PD-L2 expression, however, PD-L1 expression is not detected in most of bladder cancer cell lines except parental T24 cell (Fig. 4D). These data imply that PD-L2 but not PD-L1 is involved in bladder cancer progression. In addition to PD-L2, there are immune checkpoint molecules (such as CD47 and CLEC4G) showing positive correlation with SPHK1 among bladder cancer patients, but not seen in the others such as VTCN1, CEACAM1, and IGSF11 (Fig. S3), implying SPHK1 might have other effects on cancer immune evasion by interacting with these molecules. Meanwhile, SPHK1 manipulation in T24L, 253J-BV, 253J, and UMUC13 showed inconsistent PD-L1 expression (Fig. S4), suggesting no direct regulation of PD-L1 expression by SPHK1. To verify whether PD-L2 is regulated by SPHK1, we found that PD-L2 expression was decreased in SPHK1 knockout T24L sublines. Meanwhile, S1P treatment could overcome the decline of PD-L2 expression in SPHK1 knockout T24L clone #A. SPHK1 inhibition by FTY720 treatment could suppress PD-L2 expression as well (Fig. 4E). In the SPHK1 knockout 253J-BV sublines, we also found that PD-L2 expression is decreased in both clones. S1P treatment could overcome the decline of PD-L2 expression in SPHK1 knockout 253J-BV clone #B. SPHK1 inhibition by FTY720 treatment could suppress PD-L2 expression as well (Fig. 4E). On the other hand, the PD-L2 expression was increased under S1P treatment on 253J cell, and 253J-CA had higher PD-L2 expression than 253J-VC, which the PD-L2 expression can be further suppressed under FTY720 treatments (Fig. 4F). In the UMUC13 cells, we also found that PD-L2 expression was increased under S1P treatment on UMUC13 cells, and UMUC13-CA had higher PD-L2 expression than UMUC13-VC, which the PD-L2 expression can be further suppressed under FTY720 treatments (Fig. 4F). Then, to verify whether PD-L2 is correlated to bladder cancer cell migration and invasion, CRISPR PD-L2 knockout 253J-BV and PD-L2 OE 253J sublines were established for further studies (Fig. 4G). As the results, the cell migration and invasion abilities were decreased significantly in PD-L2 knockout 253J-BV subline (clone #C); on the other hand, the cell migration and invasion abilities were elevated in PD-L2 OE 253J subline (Fig. 4H). It shows that PD-L2 can regulate bladder cancer cell migration and invasion.

### The induction of PD-L2 expression by SPHK1 via PI3K/Akt/β-catenin signaling pathway

**Fig. 5:**
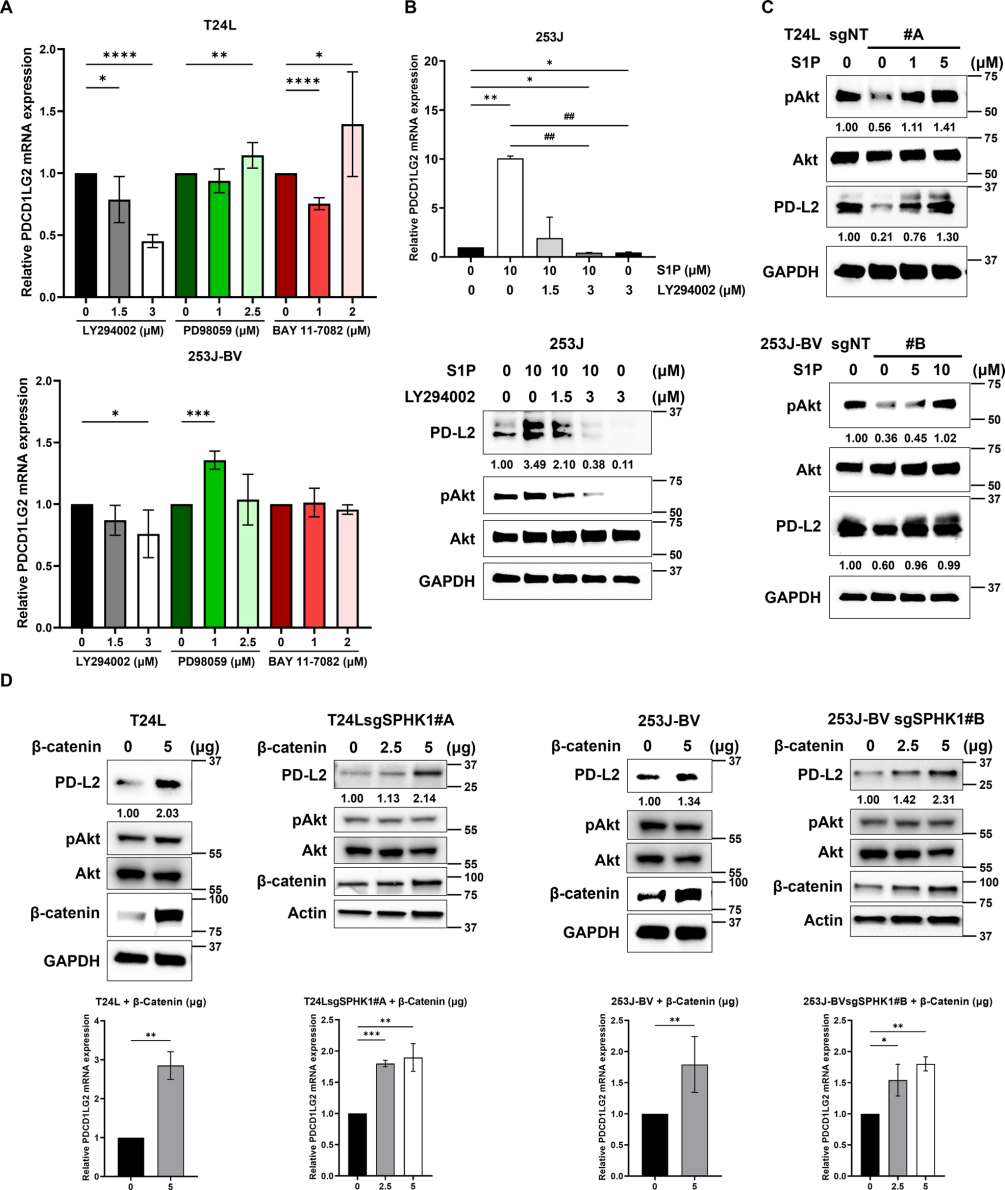
The role of PI3K/Akt/β-catenin signaling pathway in SPHK1-induced PD-L2 expression. **A** The expression of PD-L2 mRNA in T24L or 253J-BV cell lines treated with various inhibitors for PI3K/Akt, ERK, or NF-κB pathway (*n* = 3). **B** The inhibitory effect of LY294002 on PD-L2 mRNA and protein expression in 253J cells treated with S1P (*n* = 3). **C** The effect of S1P on Akt activation or PD-L2 protein levels in T24LsgSPHK1 or 253J-BVsgSPHK1 cells (*n* = 3). **D** The effect of β-catenin OE on Akt activation or PD-L2 protein levels in T24LsgSPHK1 or 253J-BVsgSPHK1 cells (*n* = 3). The data were presented as mean ± SD and statistically calculated (*p < 0.05, **p < 0.01, ^##^*p* < 0.01, ***p < 0.001, ****p < 0.0001).

SPHK1/S1P/S1PR is known to elicit several downstream pathways such as PI3K, ERK, NF-κB, etc., we examined the key pathway underlying the induction of PD-L2 gene expression. Employing different pathway inhibitors (such as PI3K/Akt-pathway [LY294002], ERK-pathway [PD98059], or NF-κB pathway [BAY 11-7082]), we found only PI3K/Akt inhibitor could suppress PD-L2 gene expression in T24L and 253J-BV cell lines (Fig. 5A). Meanwhile, PI3K/Akt inhibition suppressed PD-L2 expression in 253J cells treated with S1P (Fig. 5B). Moreover, S1P can rescue PD-L2 expression in T24LsgSPHK1#A and 253J-BVsgSPHK1#B parallel with pAkt elevation (Fig. 5C). Knowing the PI3K/Akt/β-catenin pathway as an essential signaling regulates migration, invasion, and metastasis of several cancer types cancer development [26–28], we transiently overexpressed wild-type β-catenin in both SPHK1 knockout subline T24LsgSPHK1#A and 253J-BVsgSPHK1#B to confirm whether β-catenin can restore PD-L2 expression. As the results, β-catenin overexpression rescued the downregulated PD-L2 expression in both SPHK1 knockout T24L and 253J-BV sublines while Akt activity remained unchanged, indicating SPHK1 promotes PD-L2 expression through PI3K/AKT/β-catenin-axis (Fig. 5D). Moreover, we observed that PI3K/Akt inhibition leads to PD-L2 downregulation in a dose-dependent manner for T24L and 253J-BV cell lines (Fig. S5). It shows that SPHK1 can regulate PD-L2 gene expression through Akt/β-catenin-axis in bladder cancer cell lines.

### The role of PD-L2/c-Src/Y397-FAK in SPHK1-eleicted cell migration and invasion

**Fig. 6:**
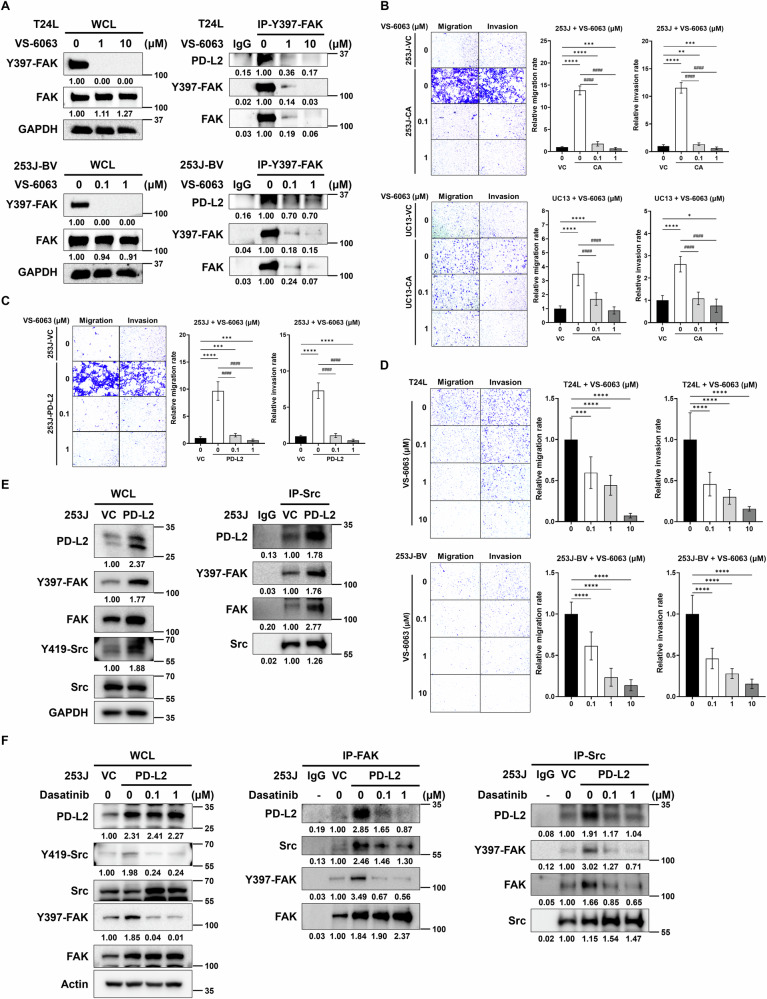
The central role of PD-L2/c-Src/Y397-FAK-complex in SPHK1-elicited cell migration and invasion of bladder cancer cells. **A** The effect of FAK inhibitor on the interaction of activated FAK protein level with PD-L2 in T24L and 253J-BV cell lines. **B** The effect of FAK inhibitor on cell migration and invasion of 253J-CA or UC13-CA sublines (*n* = 3). **C** The effect of FAK inhibitor on cell migration and invasion of 253J PD-L2 OE subline (*n* = 3). **D** The effect of FAK inhibitor on cell migration and invasion of T24L and 253J-BV cell lines (*n* = 3). **E** The protein–protein interaction between c-Src, activated FAK, and PD-L2 in 253J PD-L2 OE subline. **F** The effect of c-Src inhibitor (Dasatinib) on the formation of PD-L2/c-Src/FAK protein complex. The data were presented as mean ± SD and statistically calculated (*p < 0.05, **p < 0.01, ***p < 0.001, ****p < 0.0001, ^####^*p* < 0.0001).

We noticed that not only FAK expression but also FAK activation (i.e., Y397 phosphorylation) were elevated in T24L and 253J-BV cells compared to 253J and UMUC13 cells (Fig. S6A), in contrast, Y397-FAK and FAK expression were decreased in both SPHK1 KO T24L and 253J-BV sublines and increased in both SPHK1-CA 253J and UMUC13 sublines (Fig. S6B), which was correlated with PD-L2 levels (Fig. 4D–F). We hypothesized that the action of PD-L2 in SPHK1-elicited cell migration and invasion is mediated by the potential interaction between PD-L2 and activated FAK. Indeed, we found that FAK inhibitor (VS-6063) can abolish the protein–protein interaction ability between activated FAK and PD-L2 in T24L and 253J-BV cells (Fig. 6A). Consistently, in the presence of VS-6063, SPHK1-induced migration and invasion in both 253J and UMUC13 sublines were suppressed (Fig. 6B) as well as PD-L2-induced migration and invasion in 253J subline (Fig. 6C). On the other hand, we demonstrated the concentrations of VS-6063 used in these experiments did not significantly decrease cell viability to rule out the impact cell toxicity of VS-6063 on the results of migration or invasion (Fig. S6C). Altogether, these data indicated that FAK is the key downstream effector of SPHK1/PD-L2-elicited cell migration and invasion.

Since c-Src has been shown to play a critical role in FAK-mediated cell motility through Y397 on FAK [29]. We therefore examined whether there is an interaction between PD-L2 and c-Src, which further activate FAK. As shown in Fig. 6E, significant elevation of activated c-Src (i.e., Y419) and FAK was detected in PD-L2 OE cells, also, the interaction among c-Src, PD-L2 and activated FAK was confirmed using immunoprecipitation by c-Src antibody (Fig. 6E). On the other hand, we found that the protein–protein interaction between PD-L2 and activated FAK was attenuated by treating PD-L2 OE cells with Src inhibitor (Dasatinib) (Fig. 6F). Thus, we believe c-Src plays a key role in harnessing PD-L2 and FAK activation underlying the promoting effect of SPHK1 on bladder cancer progression.

## Discussion

Although the majority of primary bladder cancer belong to localized NMIBC, the high rate of recurrence and further invasive progression indicate the presence of aggressiveness nature of bladder cancer characterized as MIBC [6, 7]. Since the overall cost of patients for lifelong cystoscopy examination, various treatments with medication, health care services usage, life quality maintenance, and economic/productivity losses, bladder cancer becomes one of the most expensive cancers to treat [30, 31]. Also, the median survival of MIBC with distant metastases shortens to 3–6 months [11]. Due to high rate of recurrence with wide-spread disease drives an urgent need for systematic solutions for metastatic bladder cancer treatment. Currently, the chemotherapies become or are suggested for NMIBC [32], BCG-refractory NMIBC [32], localized MIBC, and advanced metastatic disease [15]. However, only half of the patients are cisplatin-eligible [13–16], and only part of the patients can be applied FGFR3 targeted therapy or immune checkpoint inhibitors (ICIs) immunotherapies for further treatments [15, 19]. Hence, finding new targeted therapeutic strategies for metastatic bladder cancer is urgent.

**Fig. 7:**
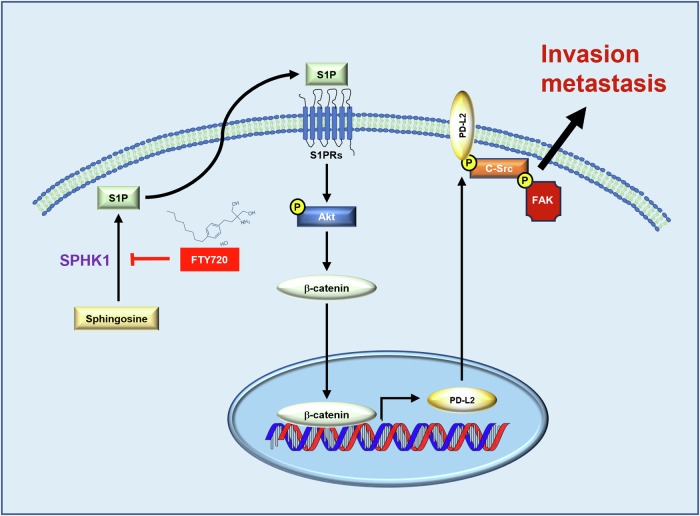
The regulator network of SPHK1 in promoting bladder cancer progression with potential targeted therapeutic strategy. In bladder cancer cells, SPHK1 phosphorylates sphingosine into S1P and then stimulated its receptors activation for further PD-L2 gene expression through Akt/β-catenin-axis. Furthermore, elevated-PD-L2 facilitates c-Src/FAK complex for promoting bladder cancer migration, invasion, and metastasis. Meanwhile, the clinically FDA-approved SPHK1 inhibitor, FTY720, attenuates the SPHK1-elicited cancer metastasis, suggesting that SPHK1 could be a potential target as novel target therapy for metastatic bladder cancer treatment.

Recently, SPHK1 has been found involved in metabolism modulation and cancer development. Several studies demonstrate that sphingosine can be phosphorylated by sphingosine kinase type 1 & 2 (SPHK1/2) and becomes S1P for engaging different cell functions, including cell proliferation, survival, migration, invasion, and metabolism. These effects are mediated through different signaling pathways such as PI3K/Akt, ERK1/2, NF-κB, STATs, Rho/ROCK, or RAS by stimulating S1PRs. Furthermore, it has been found that S225 phosphorylation on SPHK1 is vital for its enzymatic activity and function [33]. S1P functions in paracrine and autocrine phospholipids for modulating many biological activities and is involved in several metabolic syndromes; meanwhile, the ceramides/S1P ratio may affect further disease development, including cancer [34, 35]. Hence, SPHK1/2 is the master regulator for S1P production, it has been associated with the development of many types of cancer. In 2017, Liu et al. found SPHK1-induced EMT through CDH1/E-cadherin lysosomal degradation in hepatocellular carcinoma [36]. In 2019, Acharya et al. demonstrated SPHK1 promotes triple-negative breast cancer metastasis through NFκB/FSCN1-axis [37]. In 2022, Bhadwal et al. demonstrated SPHK1 is increased in tumor tissues in 31 breast cancer patients; in addition, invasion-related MMP9 and MMP2 expression and drug-resistance-related ABCC1 and ABCG2 expression are correlated with SPHK1 expression [38] and Zhang et al. demonstrated the suppressive effect of SPHK1 inhibitors such as compound “28” and PF-543 on lung metastasis of triple-negative breast cancer [39]. In 2021, Wu et al. demonstrated SPHK1 promotes colorectal cancer metastasis through paxillin [40]. Recently, we found SPHK1 promotes prostate cancer metastasis and neuroendocrine prostate cancer (NEPC) development in which FTY720 (Fingolimod), an FDA-approved SPHK1 inhibitor for multiple sclerosis, exhibits significant therapeutic efficacy using pre-clinical NEPC xenograft models [24, 41, 42]. Despite of Meng et al. found that increased SPHK1 expression is associated with poor prognosis in bladder cancer [21] and Qin et al. demonstrated SPHK1 contributes to cisplatin resistance through NONO/STAT3-axis in bladder cancer [43], the mechanism of action of SPHK1 in bladder cancer progression is poorly understood. In this study, we performed bioinformatic analyses of bladder cancer database and unveiled significant high expression of SPHK1 but not SPHK2 with clinical BS subtype [44, 45], a typically invasive cancer, or LI subtype [44, 45] that is associated with chemo resistant. Similarly, elevated SPHK1 expression is associated with metastatic sublines generated from two bladder cancer cell lines (T24 and 253J). We further demonstrated that the functional role of SPHK1 in promoting in vitro cell migration/invasion and in vivo lung metastases. Noticeably, we found PD-L2 induction by SPHK1/S1P/S1PR is mediated by Akt/β-catenin signaling pathway and PD-L2 appears to be a key downstream effector for the mechanism of action of SPHK1 (Fig. 7).

Currently, several new ICIs have been developed to target PD-1 or PD-L1 that is known as a ligand to activate PD-1-elicited T cell exhaustion [15, 46–51] in many cancer types [52, 53] including bladder cancer. Despite of a similar functional role of PD-L2, another ligand to PD-1, in immune evasion of cancer [54–56], there are a few findings regarding additional activities of PD-L2 on cancer progression. In 2022, Takamochi et al. found a clinical association of PD-L2-positive lung adenocarcinoma (LUAD) with biologically aggressive characteristics based on PD-L2 expression status from 980 surgically resected LUAD specimens [57]. Also, it has been found that PD-L2 expression is associated with a poor prognosis in patients’ cancer tissues, such as hepatocellular carcinoma [58], and esophageal cancer [59]. Furthermore, several studies demonstrate an association between PD-L2 and metastases of oral squamous cell carcinoma after cisplatin treatment [60], head and neck squamous cell carcinoma [61], or osteosarcoma [62]. Mechanistically, PD-L2 is able to activate RhoA-ROCK-LIMK2 and autophagy pathways underlying osteosarcoma invasion and metastasis [62], supporting the biologic relevance of PD-L2 with cancer progression. In our findings, we demonstrate new functional role of PD-L2 in SPHK1-elicited cell migration and invasion (Figs. 4H and 5B–D) by interacting with FAK (Fig. 6A, E, F). It is known that c-Src is able to activate FAK underlying cell motility and invasiveness [29, 63–65], we are able to demonstrate the interaction between PD-L2 with activated c-Src and FAK proteins. Moreover, FAK inhibitor (VS-6063) is able to abolish the complex formation and reduce cell migration/invasion of SPHK1 OE cells, indicating that FAK is a critical downstream effector in SPHK1-elicited cell migration and invasion (Fig. 6B, C). Meanwhile, we also rule out the role of PD-L1 in bladder cancer progression (Figs. 4D and S4). Overall, our data strengthen the pertinent role of PD-L2 in SPHK1 promoting bladder cancer progression (Fig. 7). Based on our evidence, we are optimistic about the synergy of SPHK1 inhibitor with PD-L2 inhibitor in bladder cancer treatment. Although some reports have shown the benefits of targeting PD-L2 as a drug target [55, 66], there are no promising clinically approved PD-L2 inhibitors available to date, and different strategies are needed to further develop such combination therapies. Notably, a recent phase 2 clinical trial (NCT03939234) explored a PD-L1/PD-L2 peptide vaccine in chronic lymphocytic leukemia, demonstrating the potential of PD-L2 targeting strategies even in the absence of established inhibitors [67]. This alternative approach using a vaccine supports further investigation of FTY720 combined with PD-L2 blockade for bladder cancer treatment. Therefore, developing PD-L2 targeted therapy could be beneficial for the metastatic bladder cancer treatment.

Based on the significant clinical prevalence of SPHK1 in bladder cancer progression and the functional role of SPHK1 in cell migration, invasion, and metastasis, we further explore the potential targeted therapeutic regimen of SPHK1 using clinically relevant xenograft model. An FDA-approved SPHK1 inhibitor FTY720 (Fingolimod) can effectively reduce tumor metastasis and also this regimen appeared to be biocompatible. Taken together, we conclude SPHK1 is the potent therapeutic target for developing clinical translation of metastatic bladder cancer treatment.

## Supplementary information

Supplementary Figures with legend

Original blots of WB

## Data Availability

The data are available within the article, Supplementary Information, or Original Data file. TCGA datasets used in the paper are described in the “Materials and methods” section.
