# Correction: SPHK1 promotes bladder cancer metastasis via PD-L2/c-Src/FAK signaling cascade

**DOI:** 10.1038/s41419-026-08840-9

**Published:** 2026-05-18

**Authors:** Wei-Hsiang Kao, Li-Zhu Liao, Yu-An Chen, U-Ging Lo, Rey-Chen Pong, Elizabeth Hernandez, Mei-Chih Chen, Chieh-Lin Jerry Teng, Hsin-Yi Wang, Stella Chin-Shaw Tsai, Payal Kapur, Chih-Ho Lai, Jer-Tsong Hsieh, Ho Lin

**Affiliations:** 1 Department of Life Sciences, National Chung Hsing University, Taichung, Taiwan; 2 Department of Urology, University of Texas Southwestern Medical Center, Dallas, TX USA; 3 Cancer Genome Research Center, Chang Gung Memorial Hospital at Linkou, Taoyuan, Taiwan; 4 Translational Cell Therapy Center, China Medical University Hospital, Taichung, Taiwan; 5 Division of Hematology/Medical Oncology, Department of Internal Medicine, Taichung Veterans General Hospital, Taichung, Taiwan; 6 Department of Post-Baccalaureate Medicine, College of Medicine, National Chung Hsing University, Taichung, Taiwan; 7 Department of Nuclear Medicine, Taichung Veterans General Hospital, Taichung, Taiwan; 8 Superintendent Office, Tungs’ Taichung MetroHarbor Hospital, Taichung, Taiwan; 9 College of Life Sciences, National Chung Hsing University, Taichung, Taiwan; 10 Urology and Pathology, University of Texas Southwestern Medical Center, Dallas, TX USA; 11 Department of Microbiology and Immunology, Chang Gung University, Taoyuan, Taiwan; 12 Rong Hsing Research Center for Translational Medicine, National Chung Hsing University, Taichung, Taiwan

**Keywords:** Bladder cancer, Metastasis, Lipid signalling, Targeted therapies

Correction to: *Cell Death & Disease* 10.1038/s41419-024-07044-3, published online 16 September 2024

In the originally published version of this Article, the AKT panel for the T24L group in Fig. 5D was incorrectly assembled: the AKT blot for 253J-BV cells was inadvertently used in place of the T24L panel. The authors verified the original Western blot records provided at the original submission to the Editorial Office and have replaced the panel with the correct AKT image for the T24L cell line in the “Corrected Fig. 5D”. The p-AKT/AKT blots were presented qualitatively (no densitometric quantification was performed), and this correction does not affect the results or conclusions.

**Original Fig. 5D** 
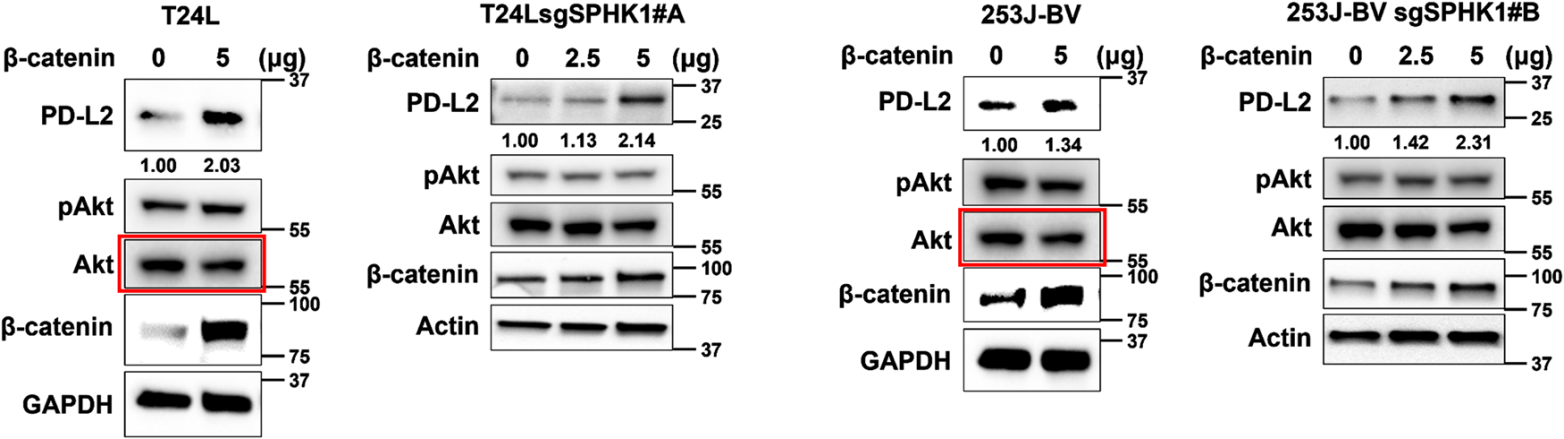


**Corrected Fig. 5D** 
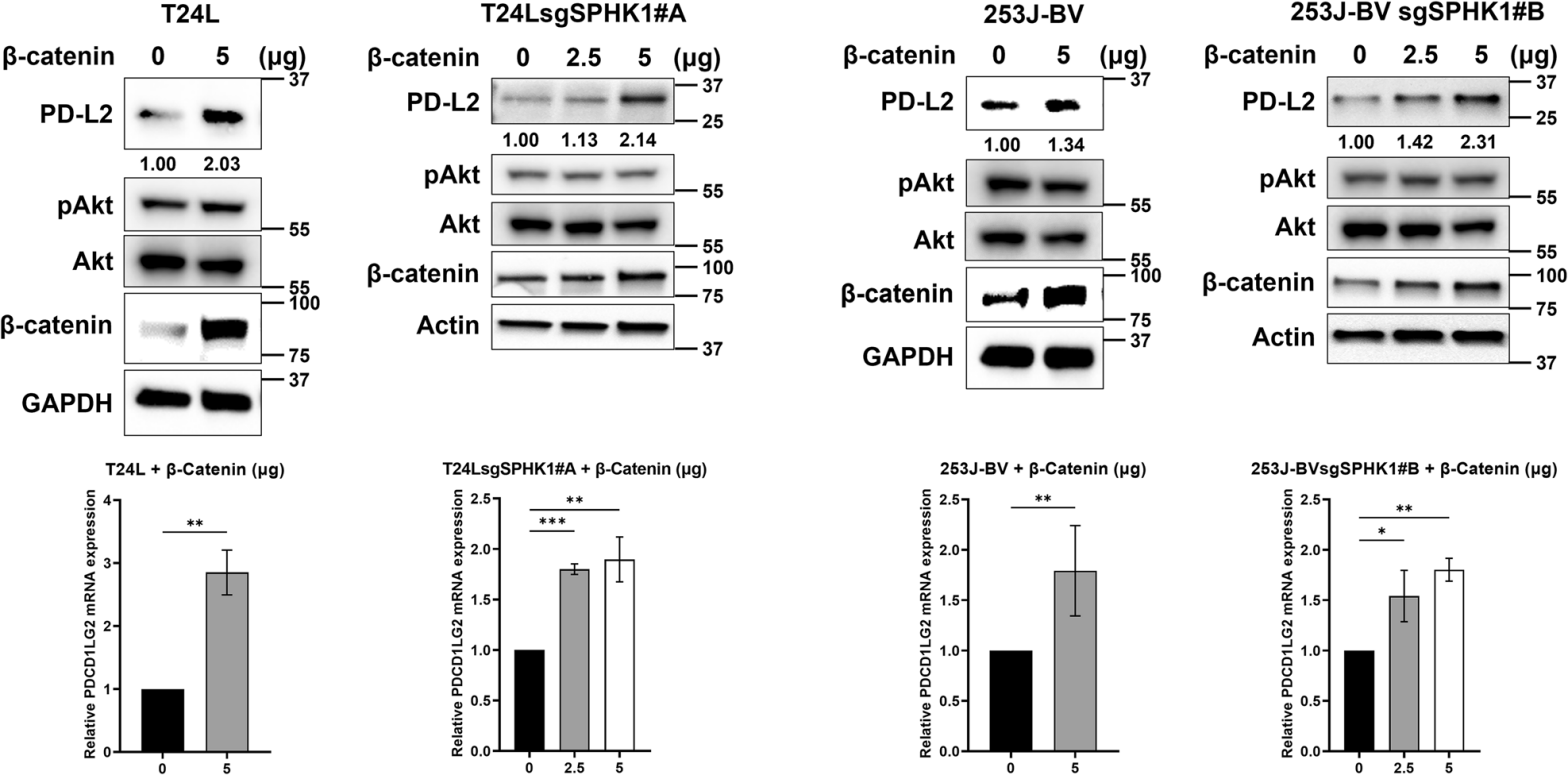


The original article has been corrected.

