# Supplementary material for: SPHK1 promotes bladder cancer metastasis via PD-L2/c-Src/FAK signaling cascade
Source: Cell Death Dis. 2024 Sep 16;15(9):678. doi: 10.1038/s41419-024-07044-3 (PMC11405731; doi:10.1038/s41419-024-07044-3)
Supplement: Supplementary file 1 — Supplementary Figures with legend [file 41419_2024_7044_MOESM1_ESM.docx]

**Supplementary Information**

**SPHK1 promotes bladder cancer metastasis via PD-L2/c-Src/FAK signaling cascade**

**Supplementary Figures 1 - 6: Page 2-8**

Wei-Hsiang Kao, Li-Zhu Liao, Yu-An Chen, U-Ging Lo, Rey-Chen Pong, Elizabeth Hernandez, Mei-Chih Chen, Chieh-Lin Jerry Teng, Hsin-Yi Wang, Stella Chin-Shaw Tsai, Payal Kapur, Chih-Ho Lai, Jer-Tsong Hsieh^#^, Ho Lin^*^

* Correspondence:

Ho Lin, Ph.D.

Department of Life Sciences

National Chung Hsing University

Taichung 40227, Taiwan

Email: [hlin@dragon.nchu.edu.tw](mailto:hlin@dragon.nchu.edu.tw)

Tel: +886-933876062

&

Science and Technology Division

Taipei Economic and Cultural Office in Australia

ACT, Australia

Email: [hlin@nstc.gov.tw](mailto:hlin@nstc.gov.tw)

Tel: +61-466833467

# Co-correspondence:

Jer-Tsong Hsieh, Ph.D.

Department of Urology

UT Southwestern Medical Center

Dallas, Texas, USA, TX75390

Email: [JT.Hsieh@utsouthwestern.edu](mailto:JT.Hsieh@utsouthwestern.edu)

Tel: +1-214-648-3988


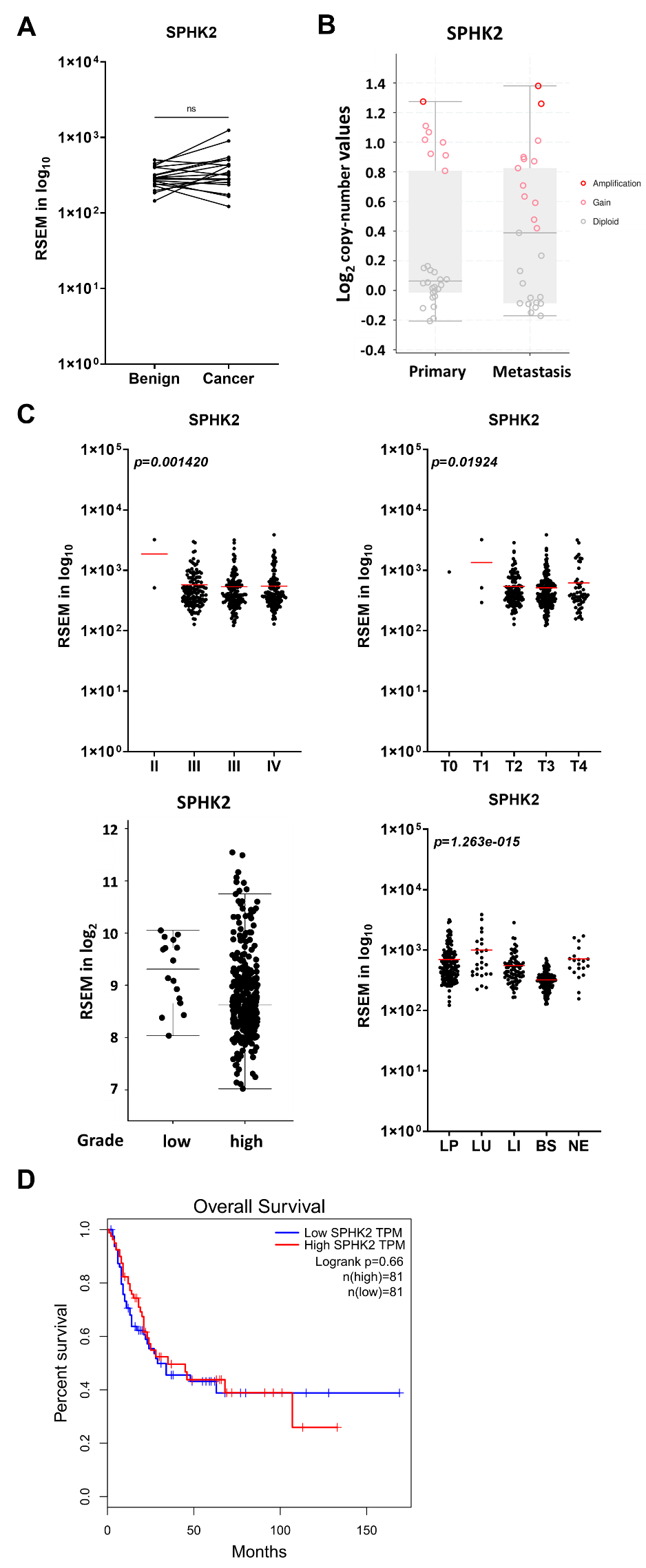
**Figure S1.**

**Figure S1. Insignificant clinical correlation of SPHK2 with bladder cancer development.** (A) Paired bladder cancer samples from the TCGA database (n = 19). (B) Primary and metastatic samples of 6 different bladder cancer TCGA dataset based on SPHK2 copy number analyzed using cBioPortal website. (C) SPHK2 gene expression in bladder cancer samples of TCGA database based on in different stage, T category in TNM classification, grade, and subtypes. (D) High and low SPHK2 expressing patients based on overall survival analyzed on GEPIA website.

**
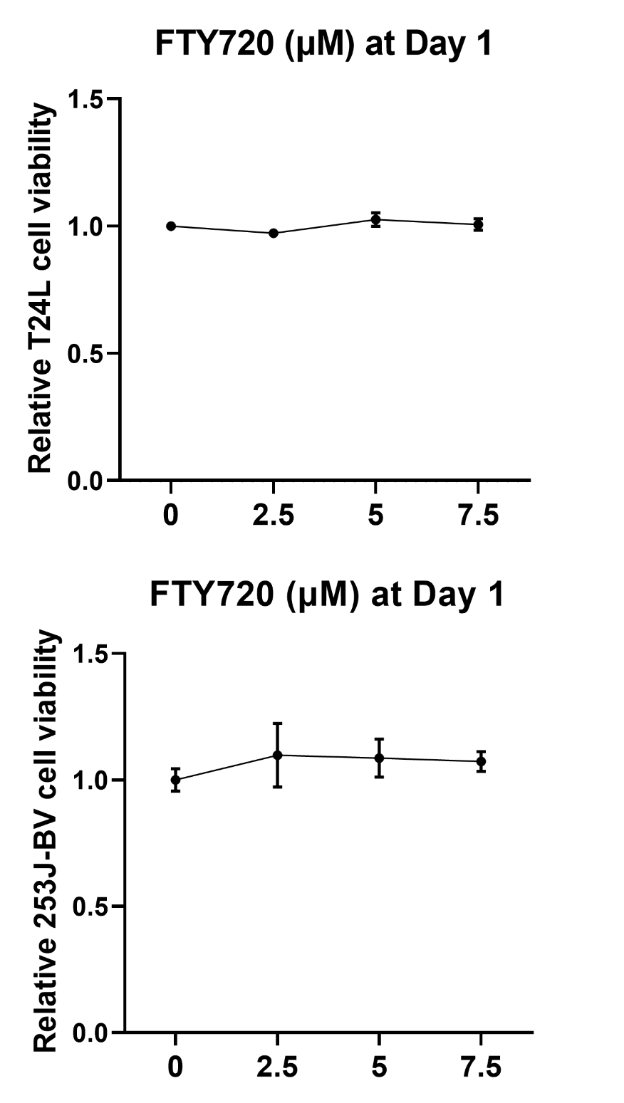
Figure S2.**

**Figure S2. The effects of SPHK1 inhibitor on the viability of T24L and 253J-BV cells.**

The MTT assay was used to determine the effects of SPHK1 inhibitor on the viability of T24L and 253J-BV cells. (n = 6)


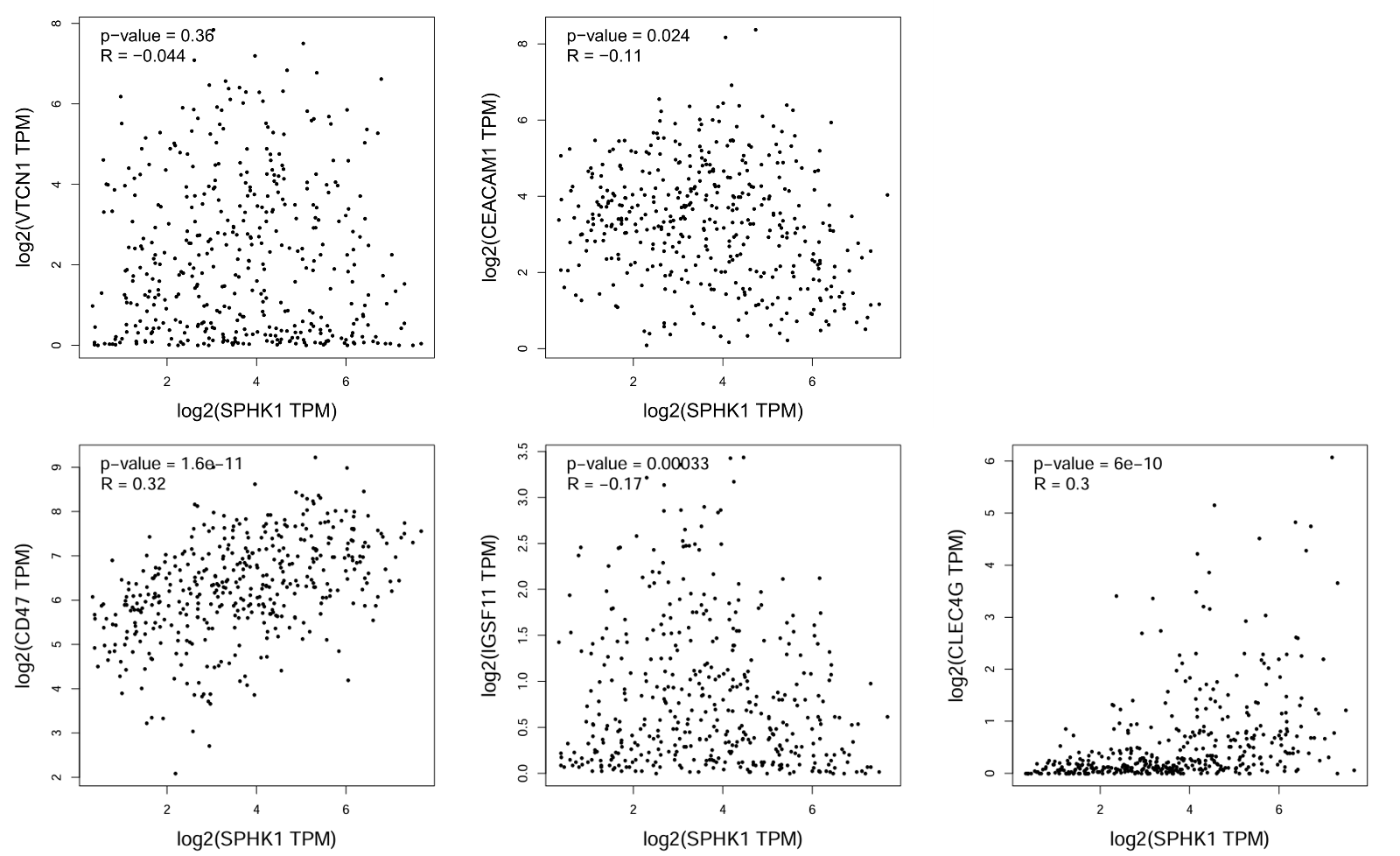
**Figure S3.**

**Figure S3. The correlation between SPHK1 and the expression of immune checkpoint molecules in bladder cancer.**

The correlation of B7-H4 (*VTCN1*), CEACAM-1 (*CEACAM1*), CD47 (*CD47*), VSIG3 (*IGSF11*) or LSECtin (CLEC4G) with SPHK1 gene expression in bladder cancer samples from TCGA database.


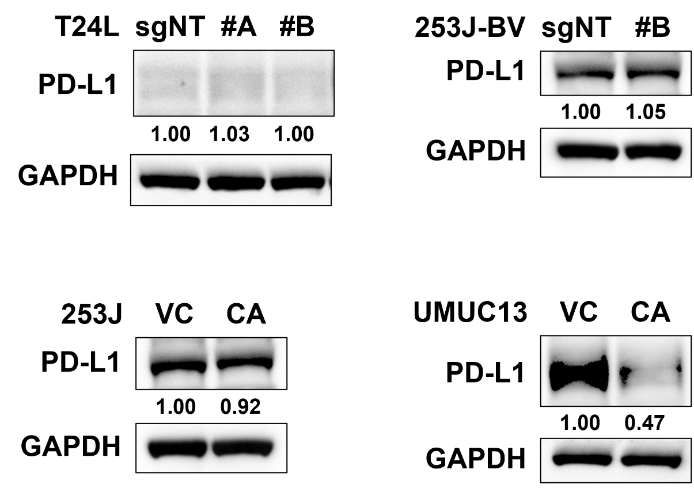
**Figure S4.**

**Figure S4. The effect of SPHK1 on PD-L1 protein expression in bladder cancer cell lines.**

The profile of PD-L1 protein expression was determined in T24LsgSPHK1, 253J-BVsgSPHK1, 253J-CA, or UC13-CA sublines.


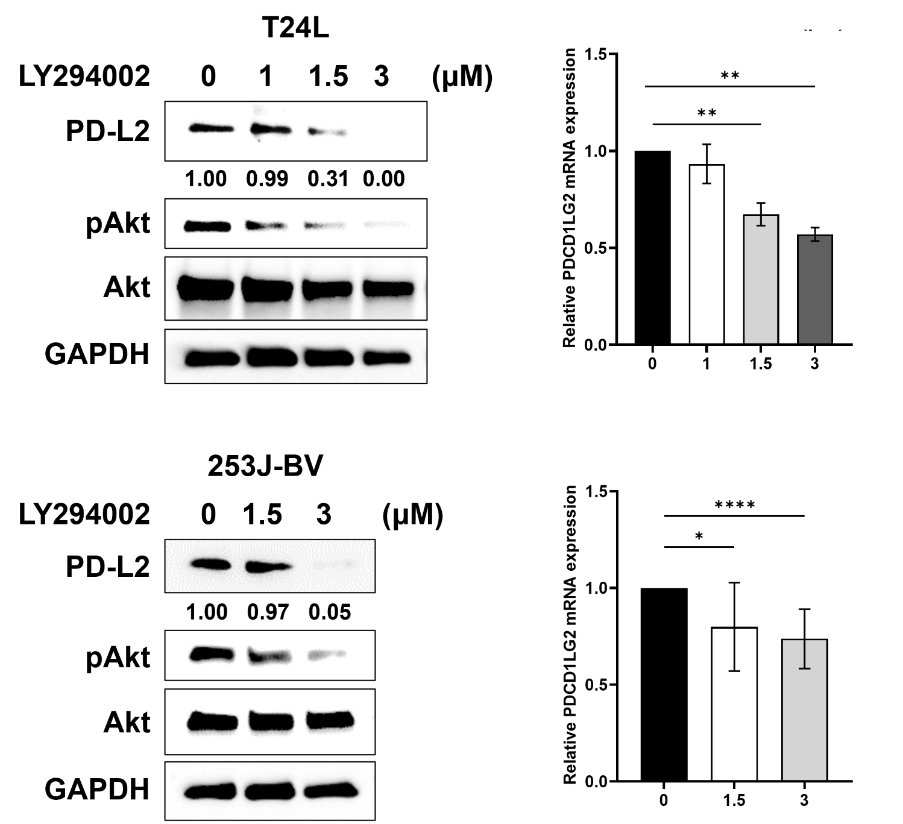
**Figure S5.**

**Figure S5. The effect of PI3K/Akt inhibitor on PD-L2 expression of T24L and 253J-BV cells.**

The expressions of PD-L2 mRNA and protein were determined in T24L or 253J-BV cell lines treated with LY294002 (n = 3) (**p*<0.05, ***p*<0.01, *****p*<0.0001).

**
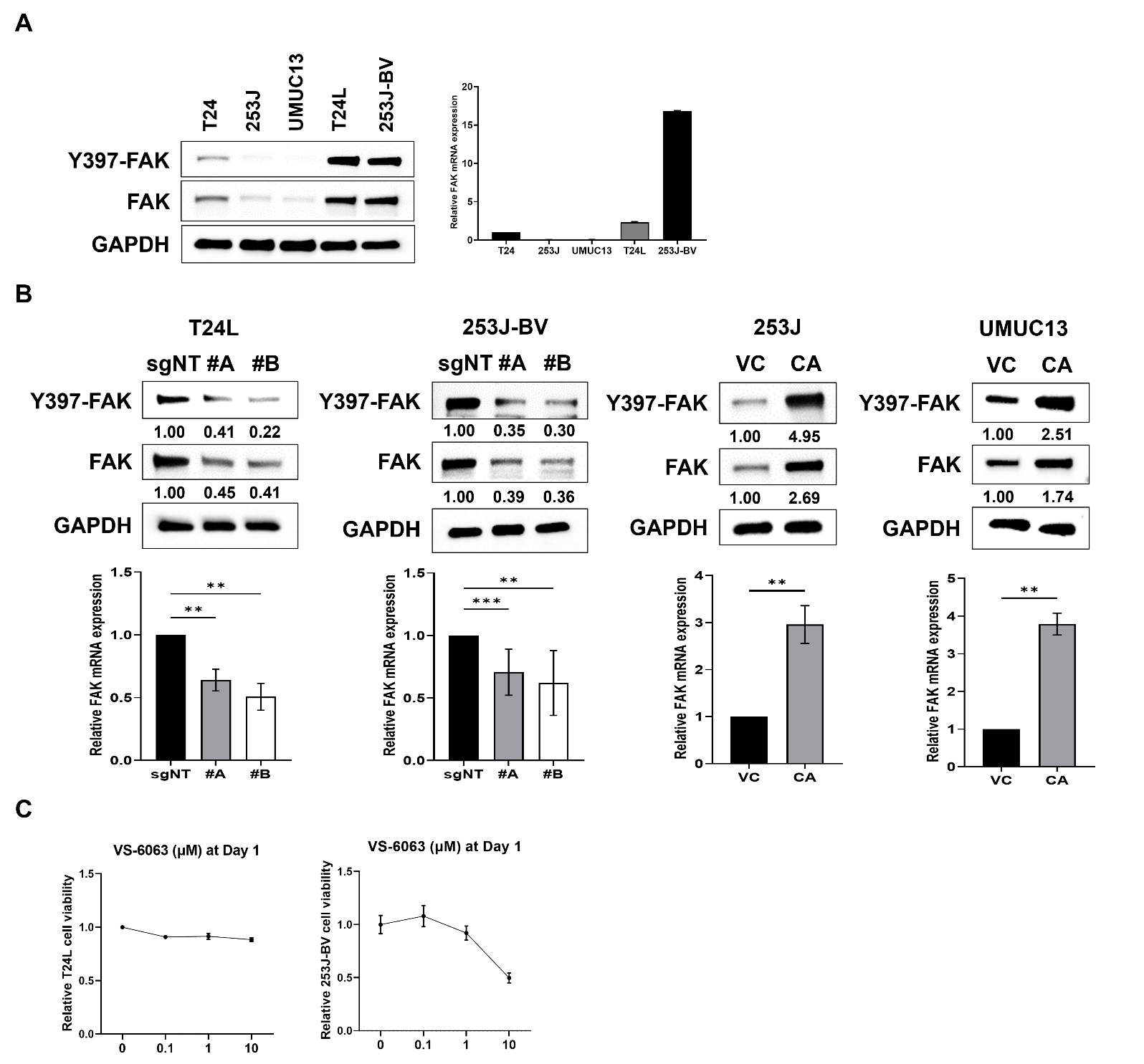
Figure S6.**

**Figure S6. The role of SPHK1 on FAK regulation in bladder cancer cell lines. (A)** The expression of FAK mRNA and protein (n = 3). (B) The effect of SPHK1 on the expression of FAK mRNA and protein (n = 3) (***p*<0.01, ****p*<0.001). (C) The effect of FAK inhibitor on cell viability of bladder cancer cell lines (n = 6).
