# Supplementary material for: SPHK1 promotes bladder cancer metastasis via PD-L2/c-Src/FAK signaling cascade
Source: Cell Death Dis. 2024 Sep 16;15(9):678. doi: 10.1038/s41419-024-07044-3 (PMC11405731; doi:10.1038/s41419-024-07044-3)
Supplement: Supplementary file 2 — Original blots of WB [file 41419_2024_7044_MOESM2_ESM.pdf]

Figure 2A

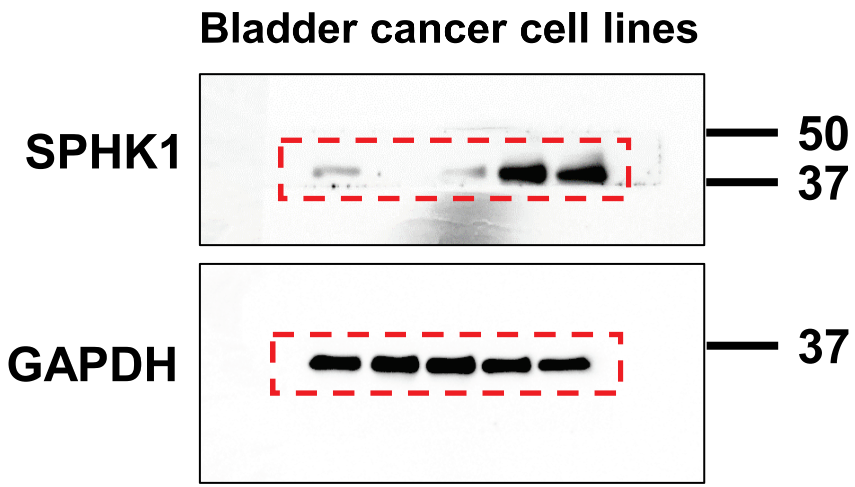

Figure 2B

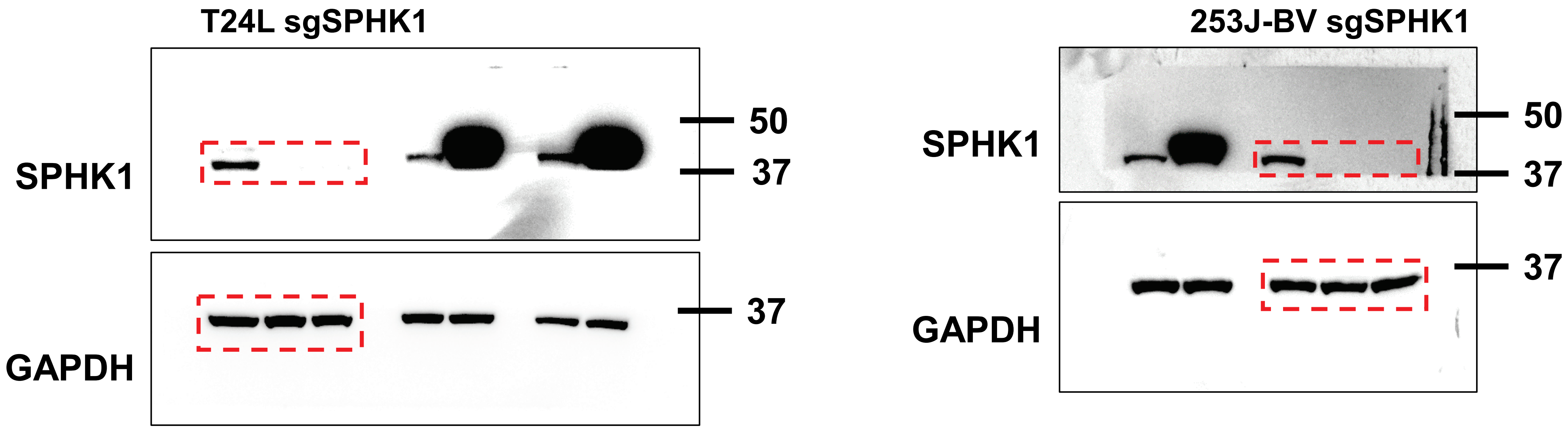

Figure 2D

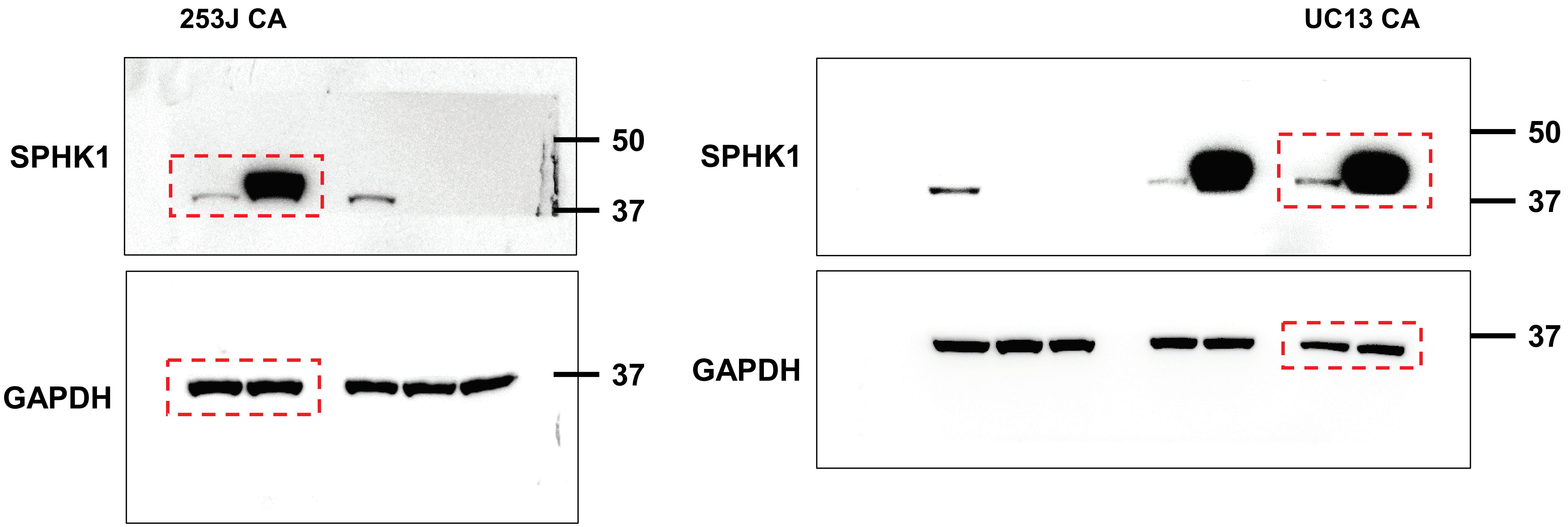

Figure 4D

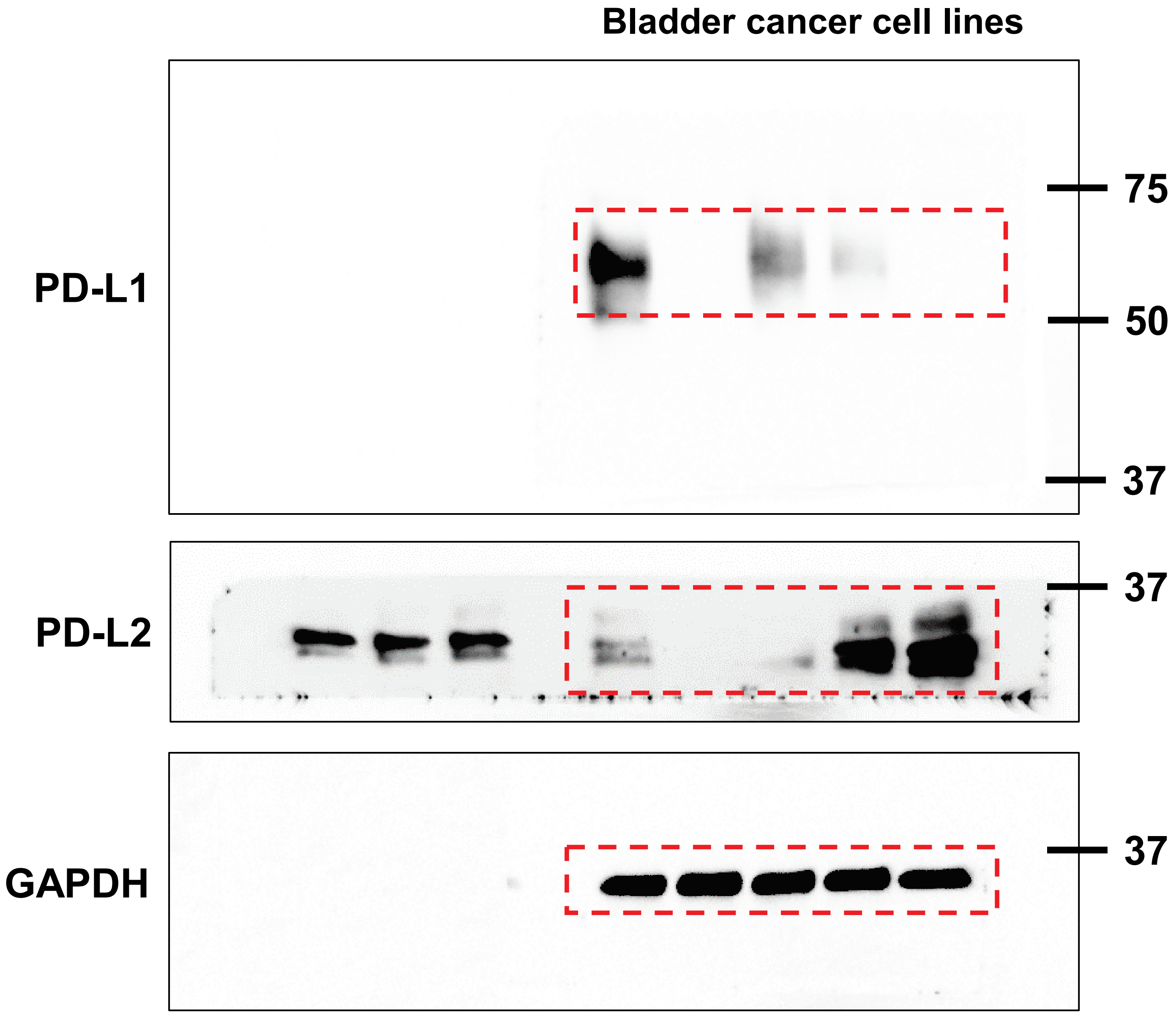

Figure 4E

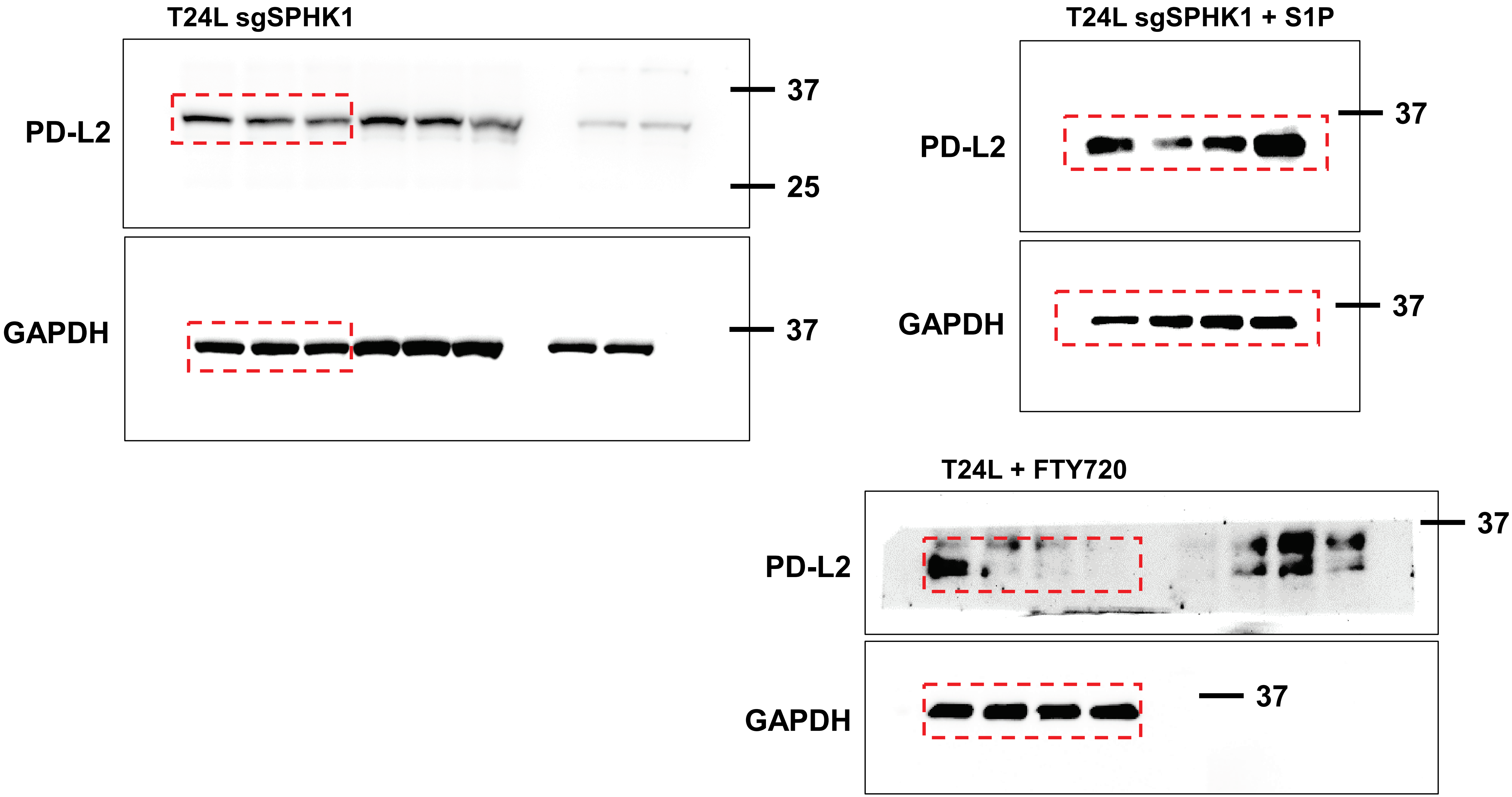
T24L sgSPHK1
PD-L2
37
25
GAPDH
37
T24L sgSPHK1 + S1P
PD-L2
37
GAPDH
37
T24L + FTY720
PD-L2
37
GAPDH
37

Figure 4E *(continue)*

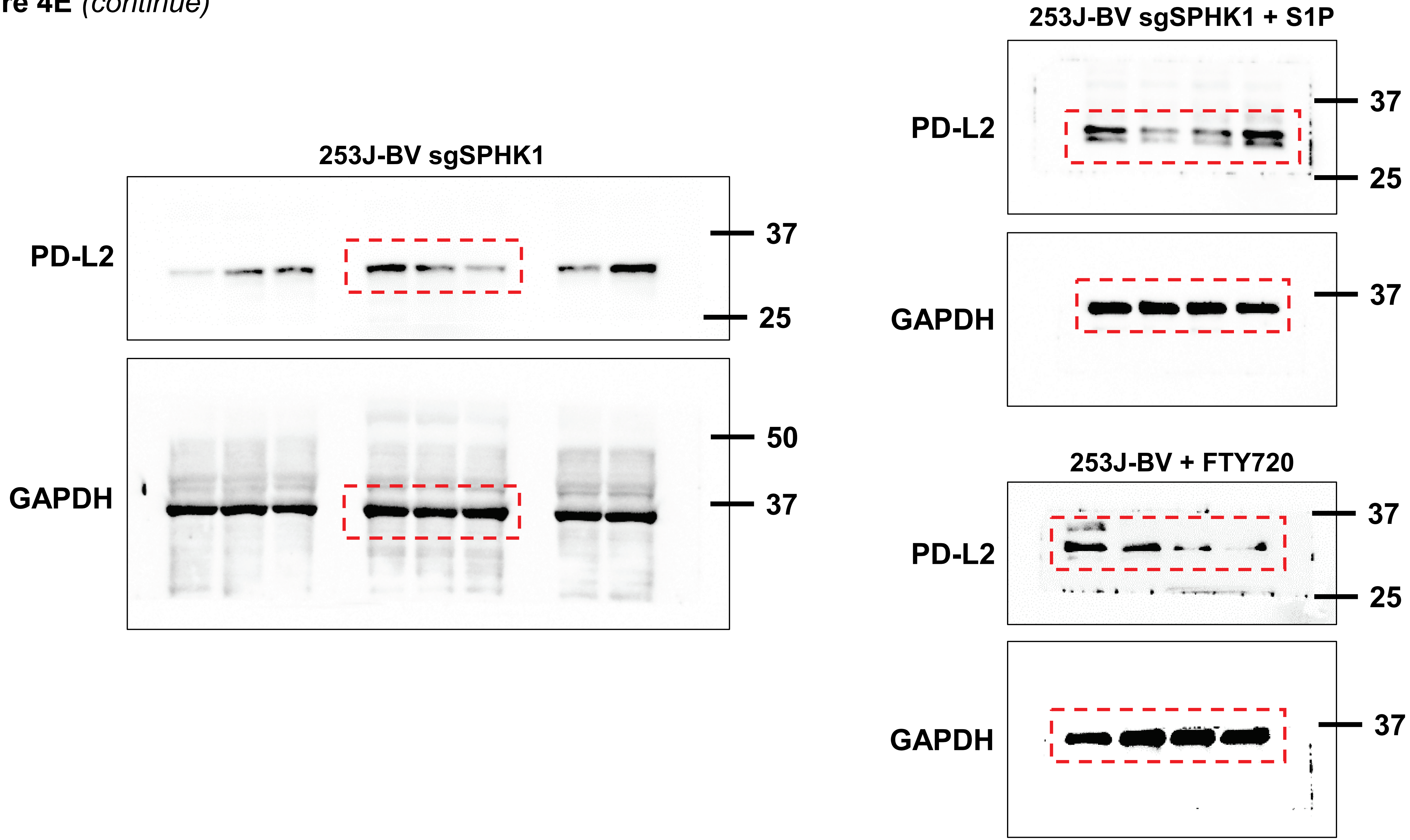

Figure 4F

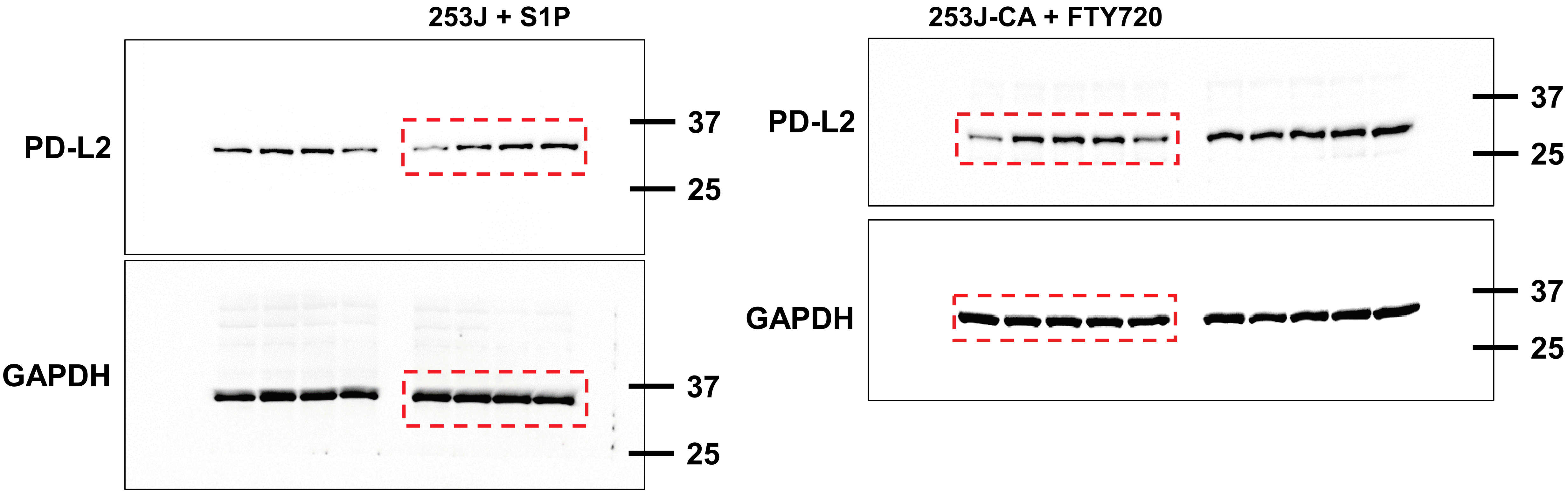
253J + S1P
PD-L2
GAPDH
37
25
37
25
253J-CA + FTY720
PD-L2
GAPDH
37
25
37
25

Figure 4F *(continue)*

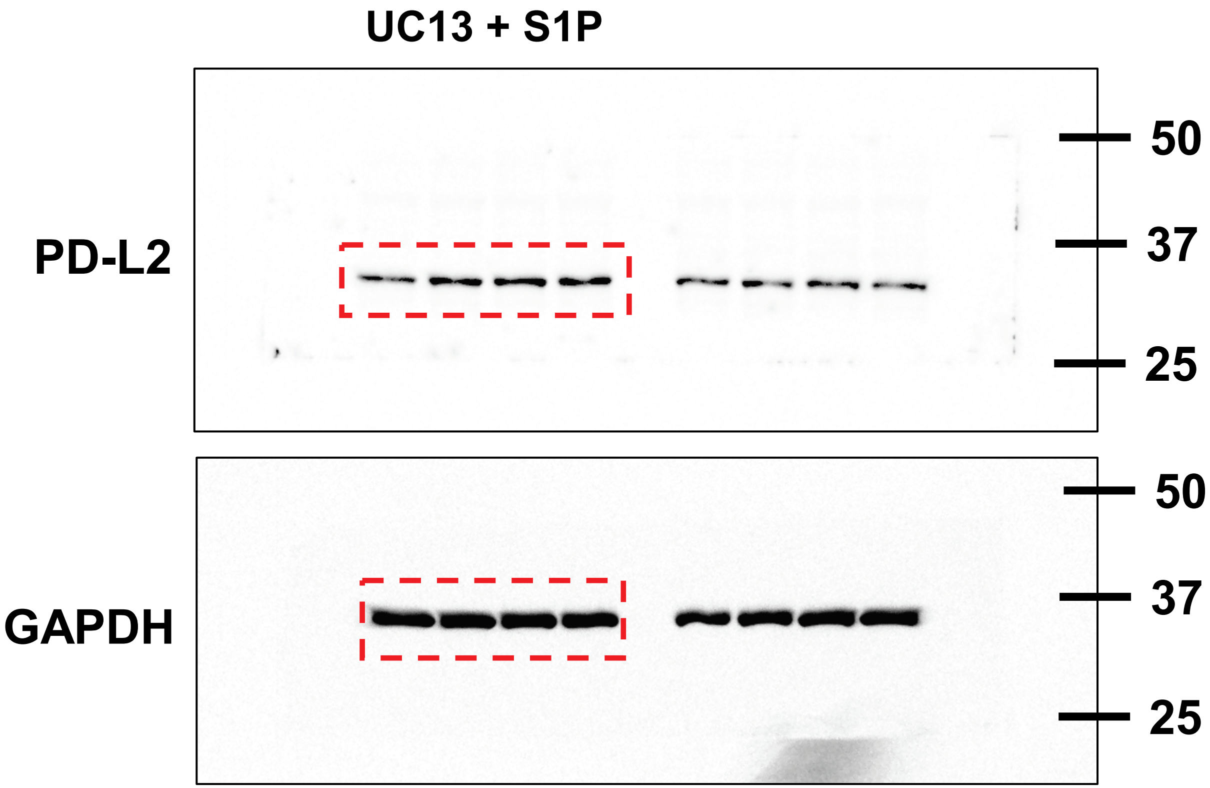

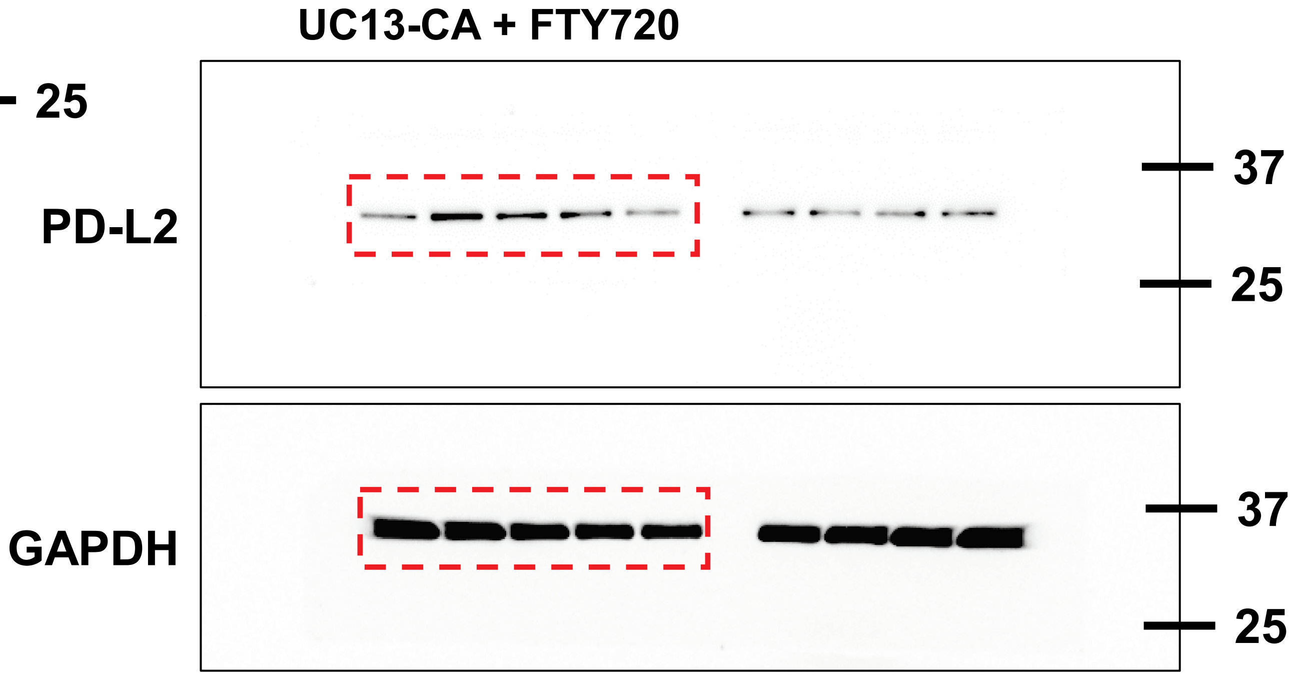

Figure 4G

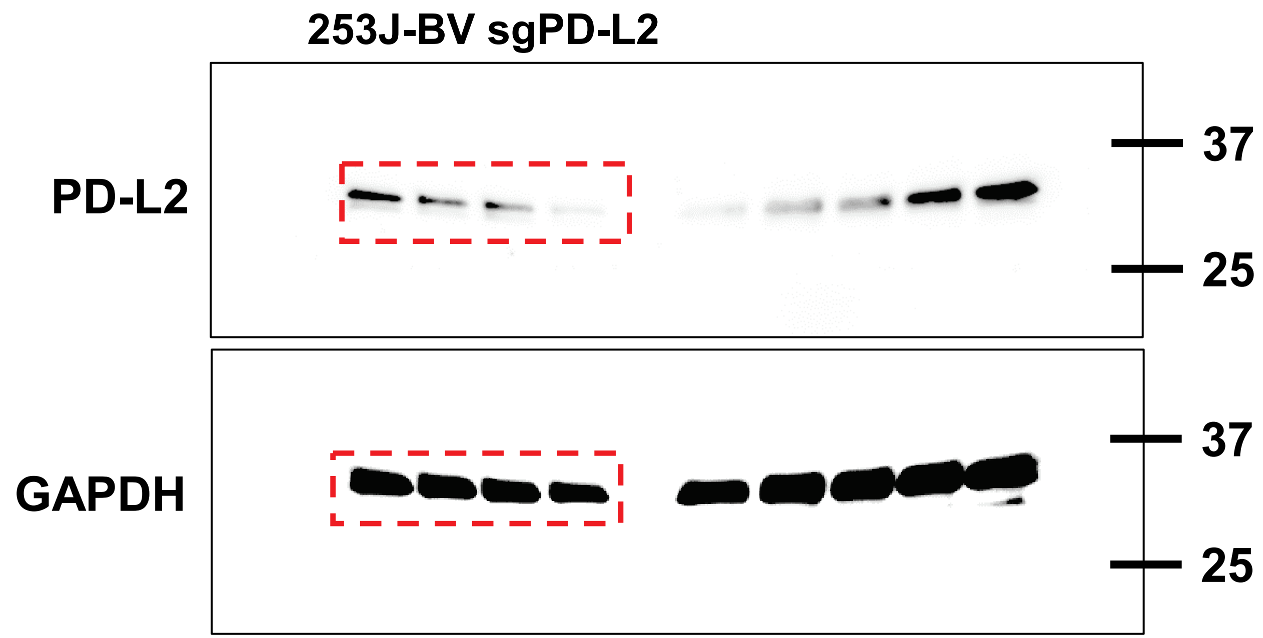
253J-BV sgPD-L2
PD-L2
GAPDH
37
25
37
25

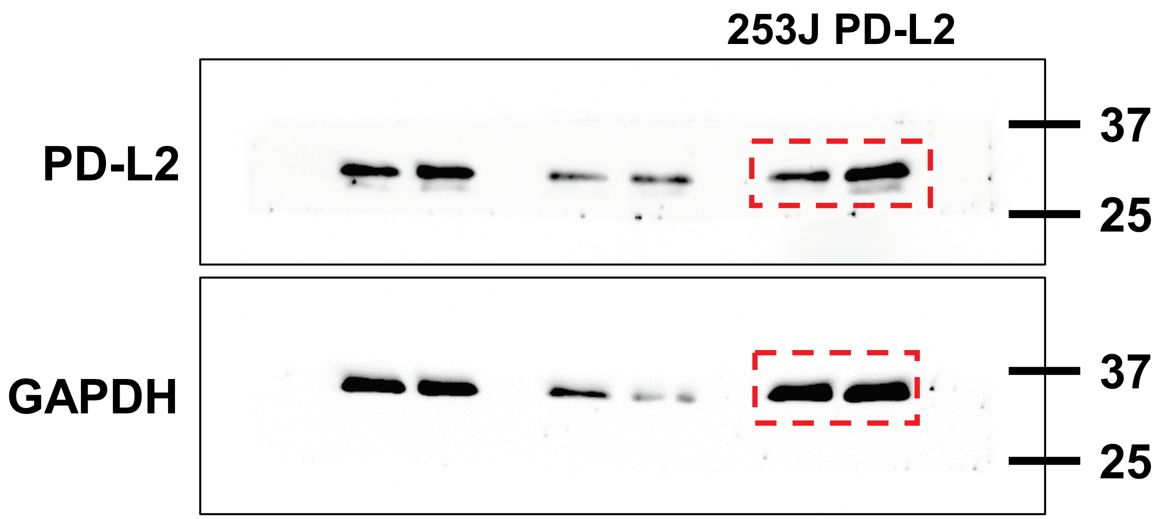
253J PD-L2
PD-L2
GAPDH
37
25
37
25

Figure 5B

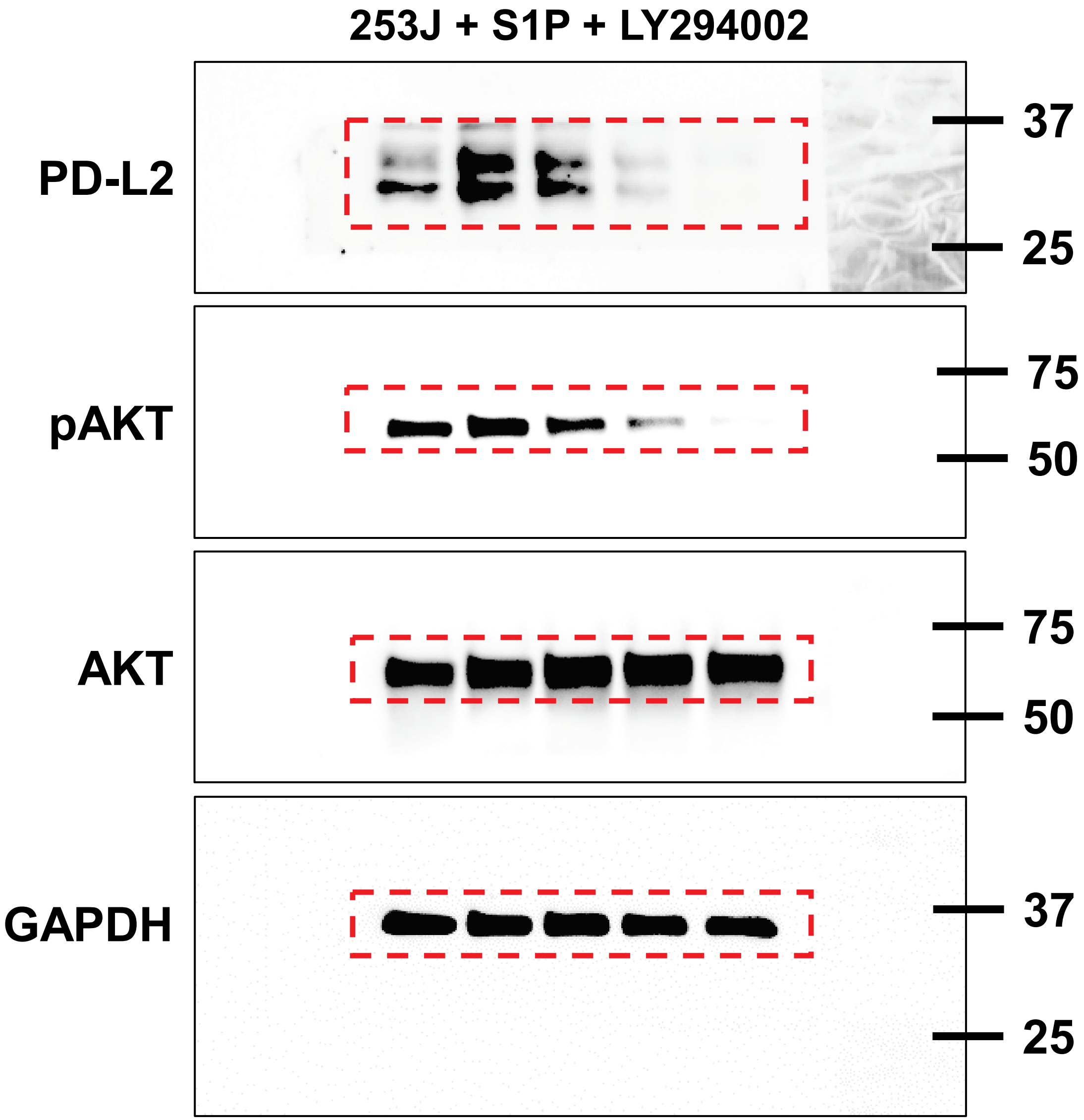

Figure 5C

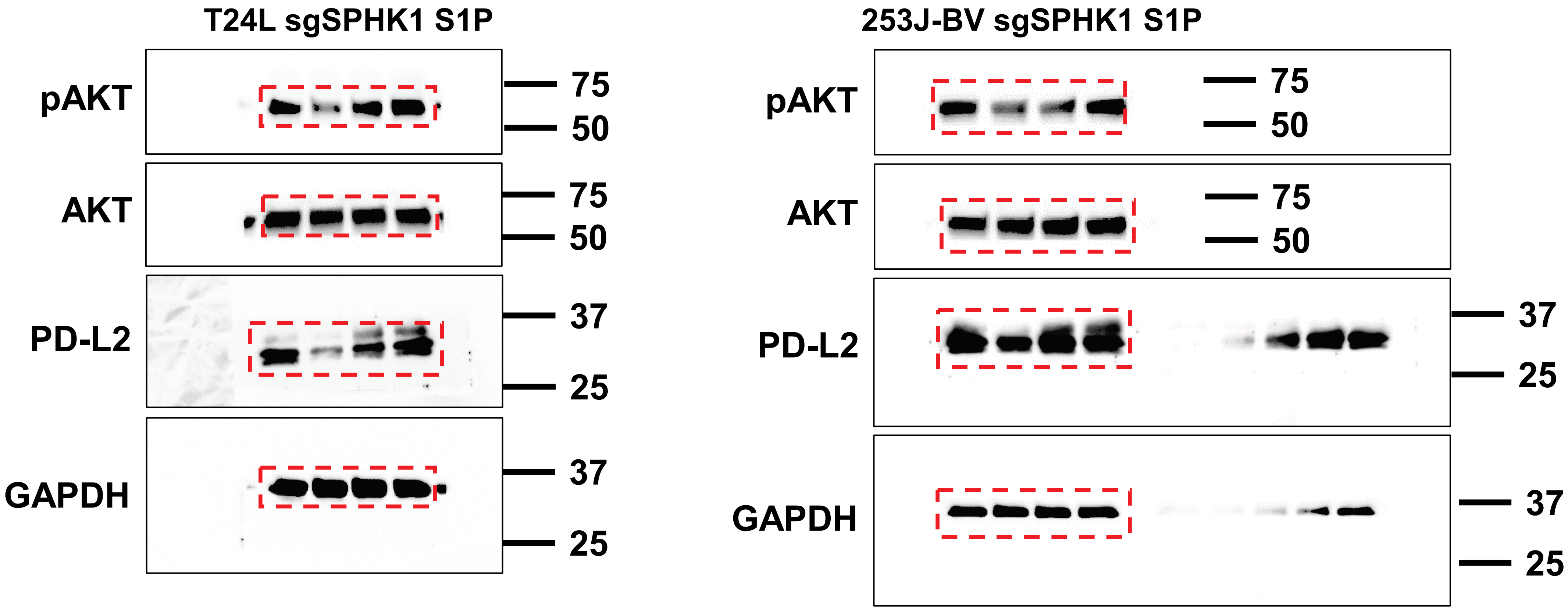

T24L sgSPHK1 S1P
pAKT
75
50
AKT
75
50
PD-L2
37
25
GAPDH
37
25
253J-BV sgSPHK1 S1P
pAKT
75
50
AKT
75
50
PD-L2
37
25
GAPDH
37
25

Figure 5D

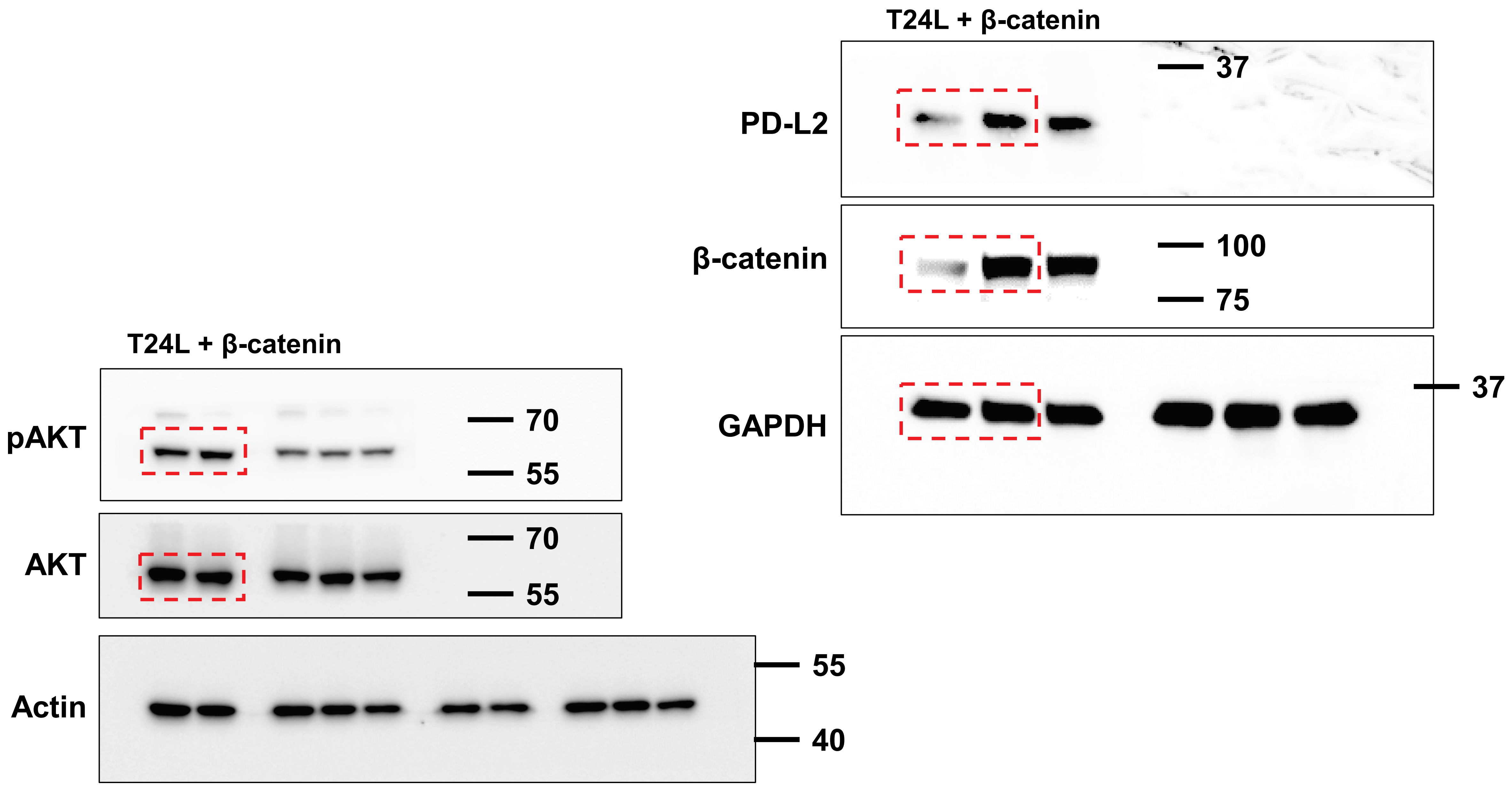

T24L + β-catenin
PD-L2
37
β-catenin
100
75
GAPDH
37
T24L + β-catenin
pAKT
70
55
AKT
70
55
Actin
55
40

Figure 5D *(continue)*

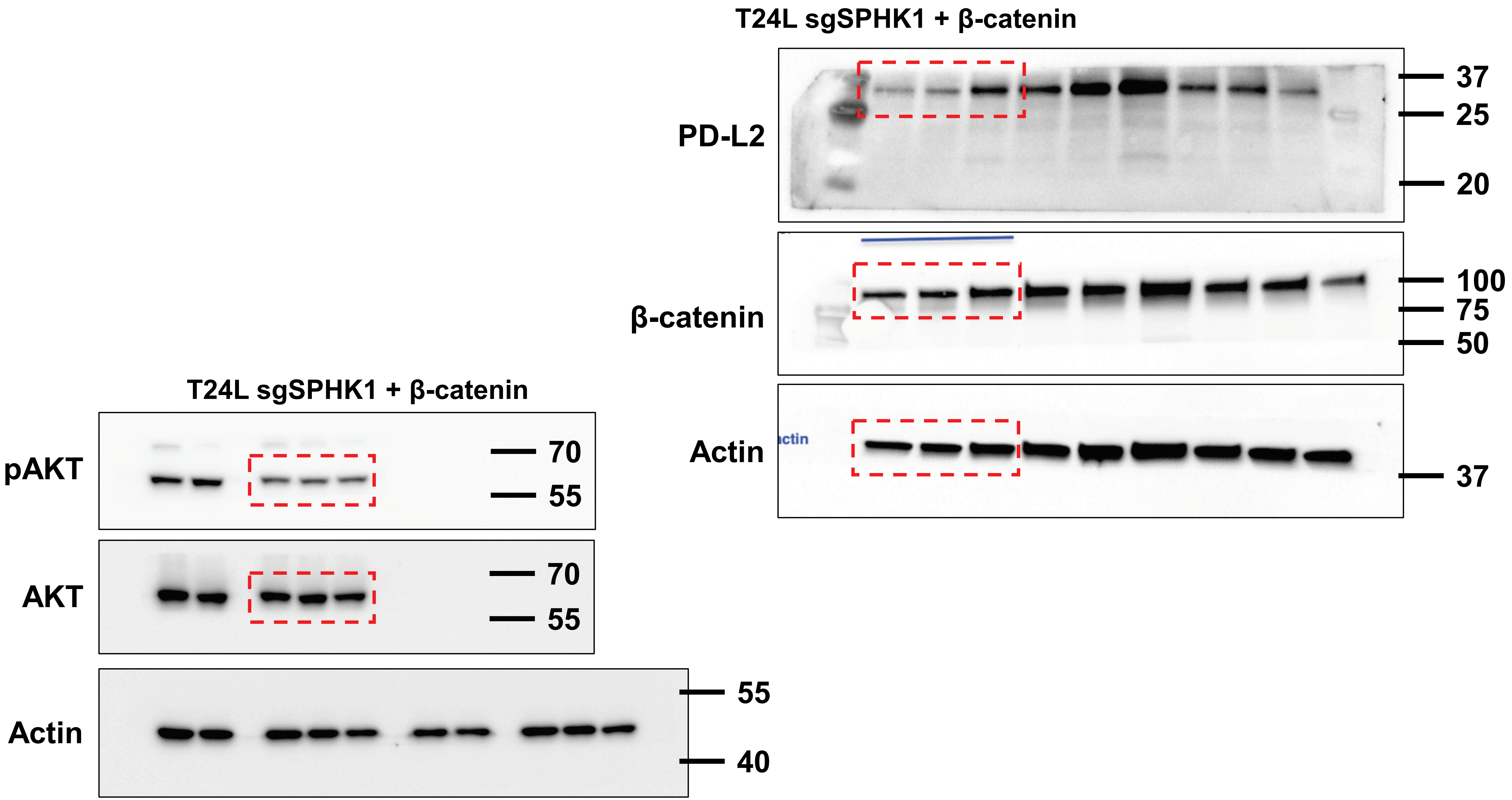

Figure 5D *(continue)*

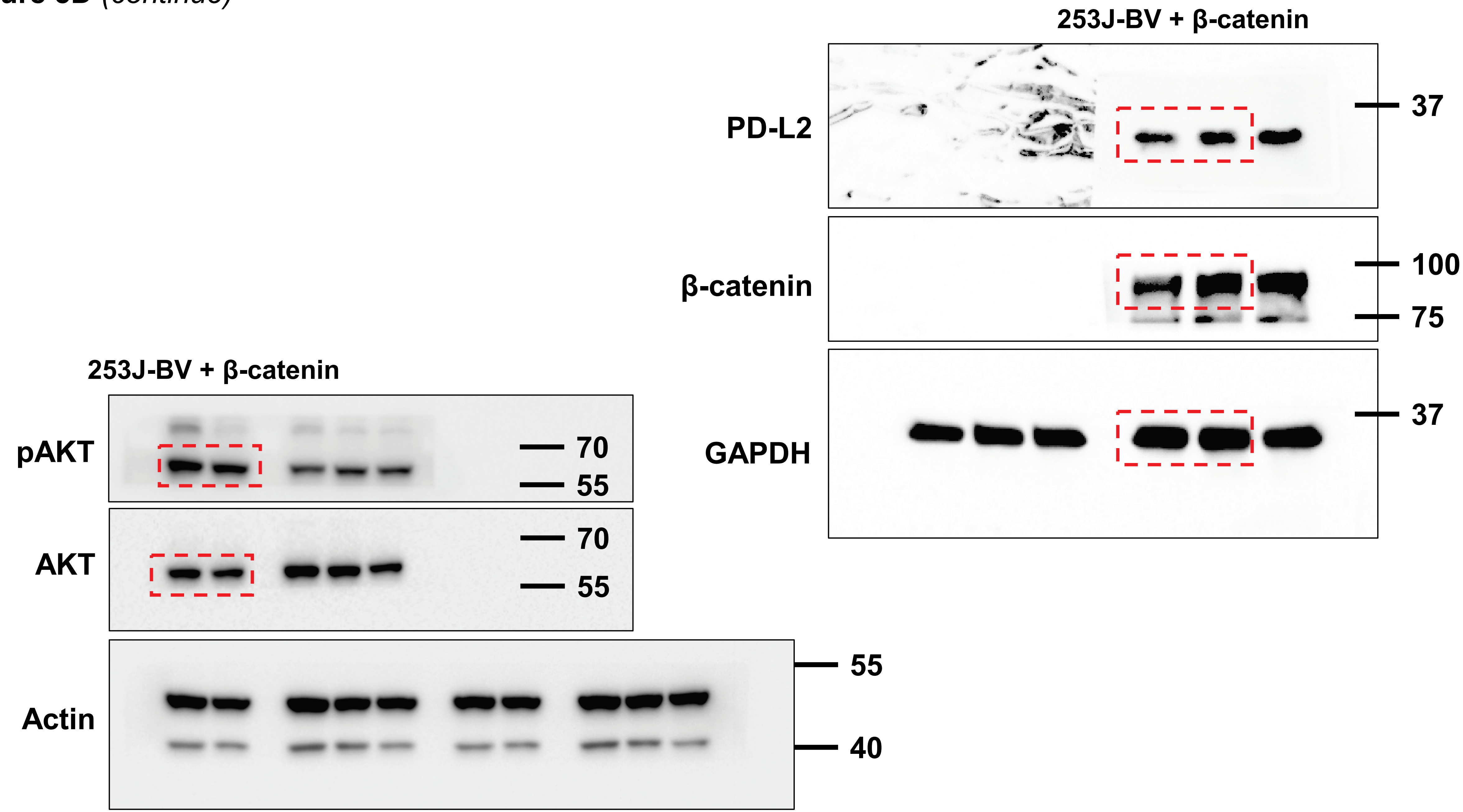

Figure 5D *(continue)*

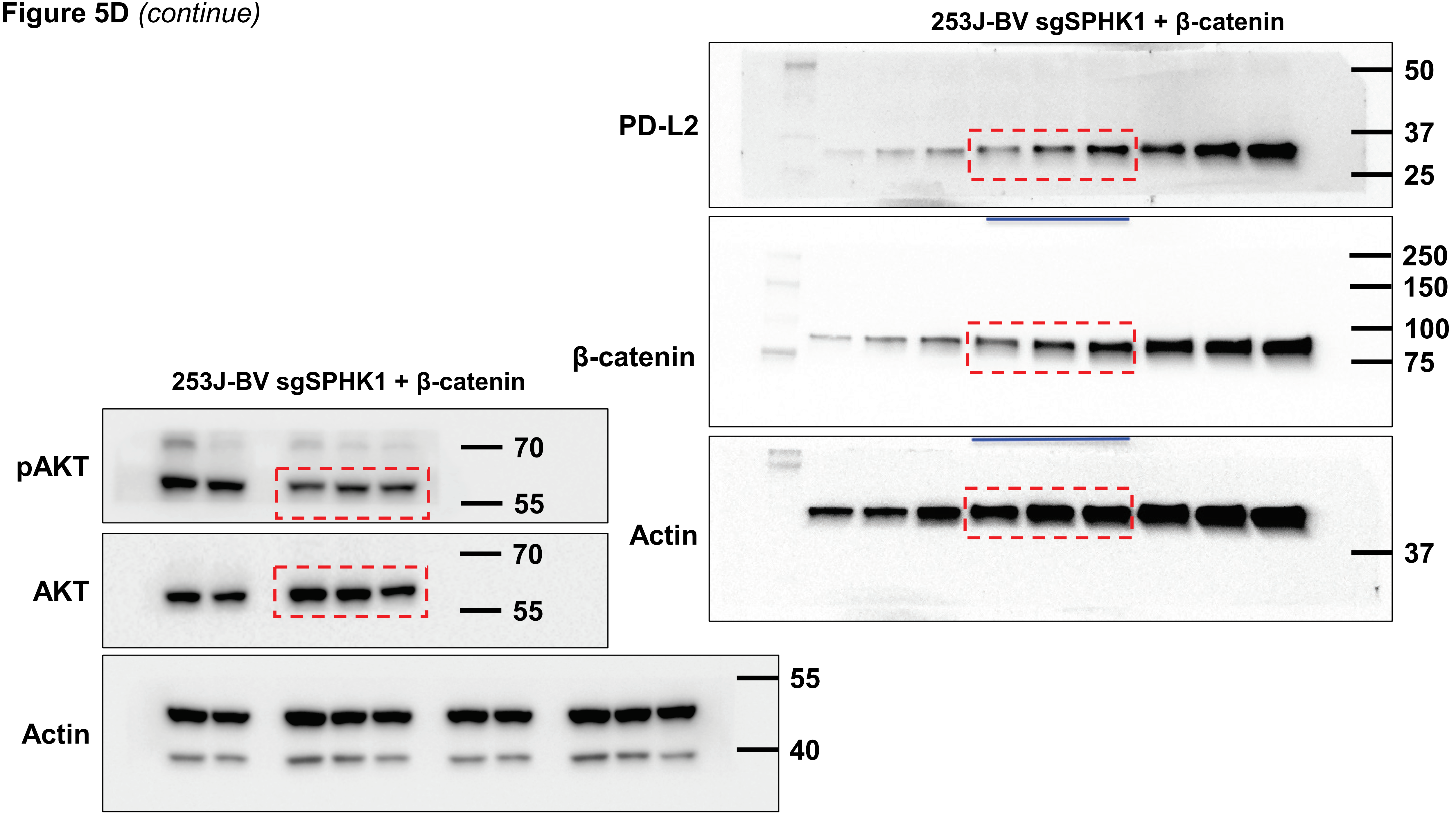

Figure 6A

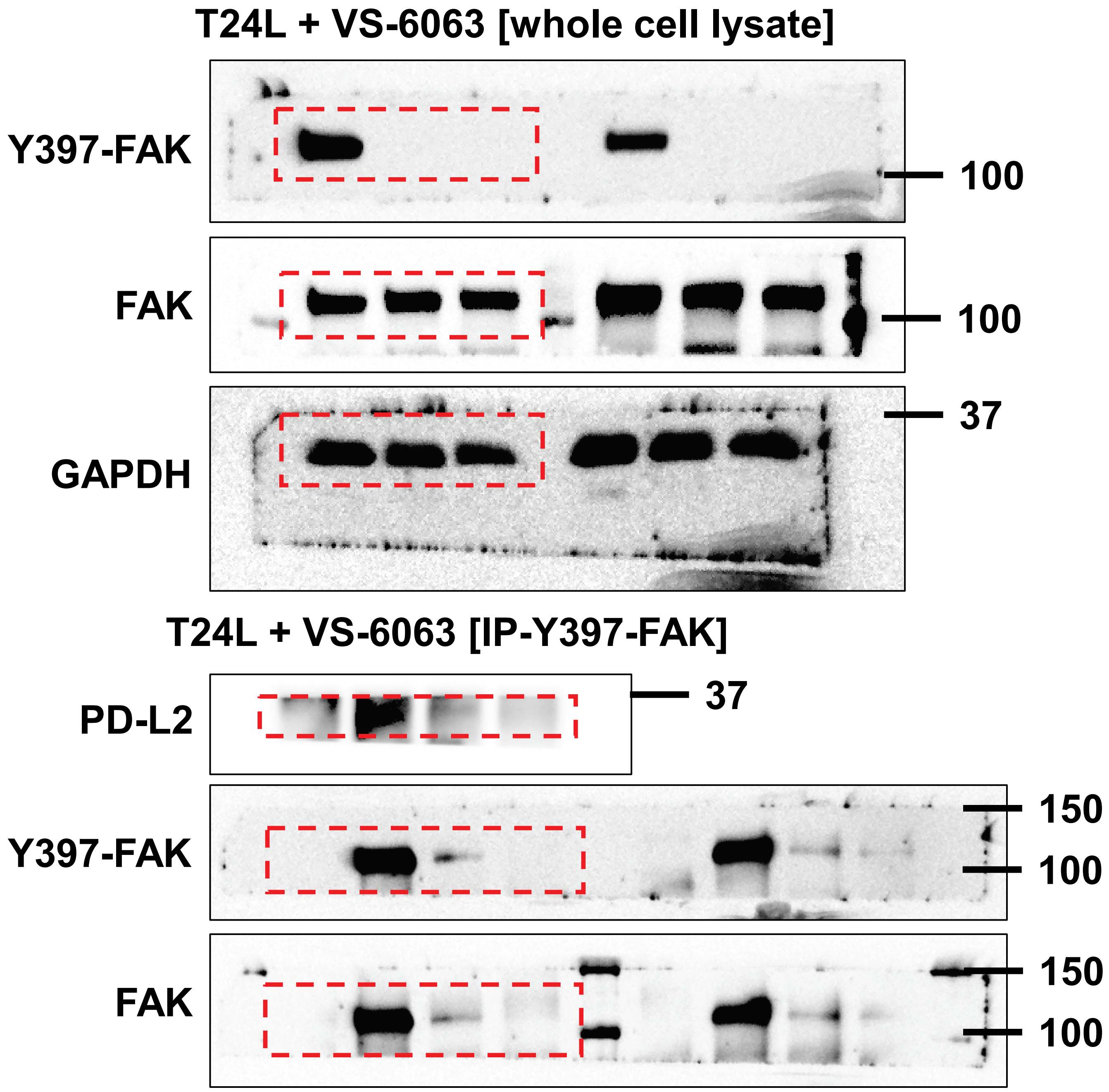
T24L + VS-6063 [whole cell lysate]
Y397-FAK
100
FAK
100
GAPDH
37
T24L + VS-6063 [IP-Y397-FAK]
PD-L2
37
Y397-FAK
150
100
FAK
150
100

Figure 6A *(continue)*

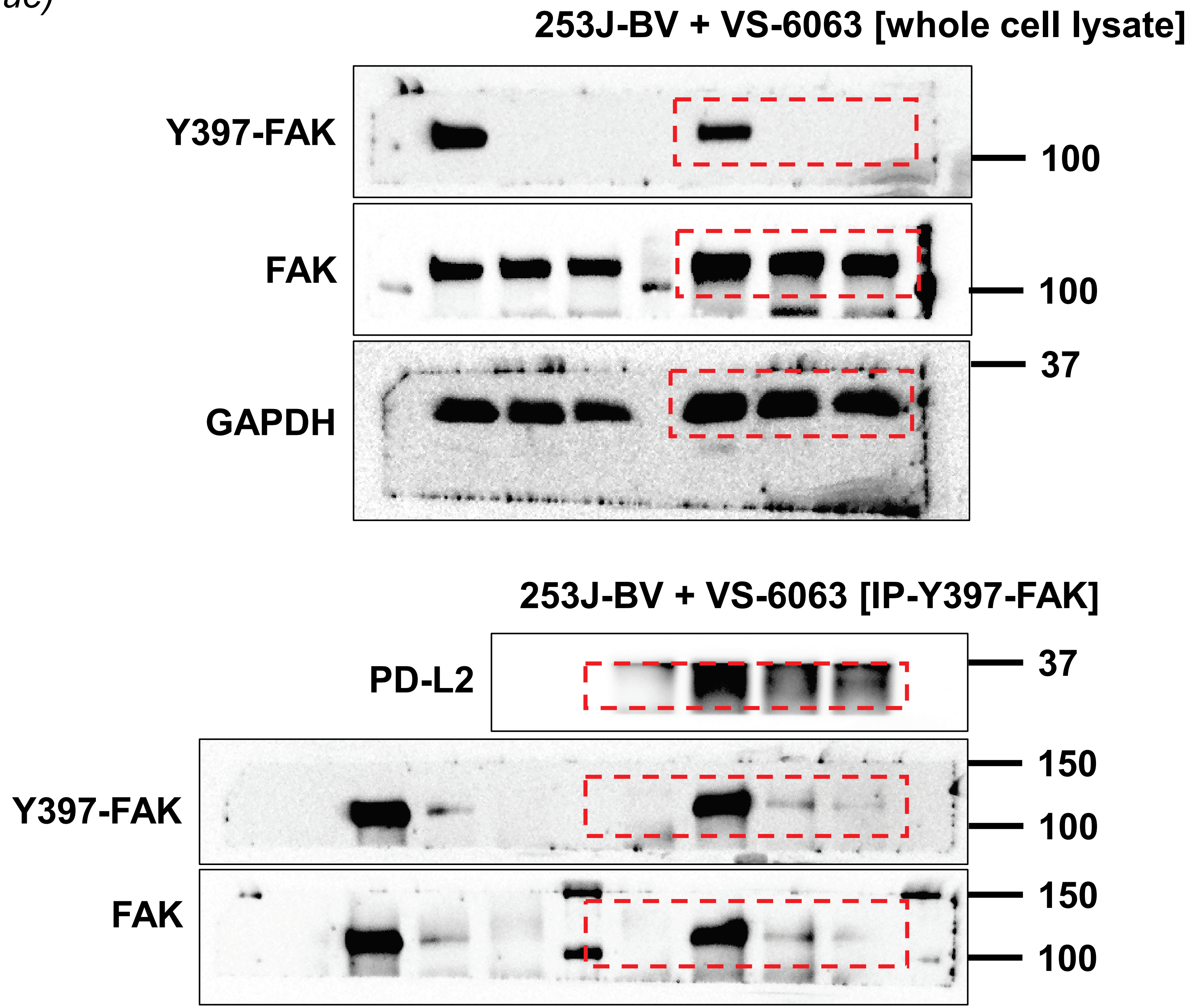

Figure 6E

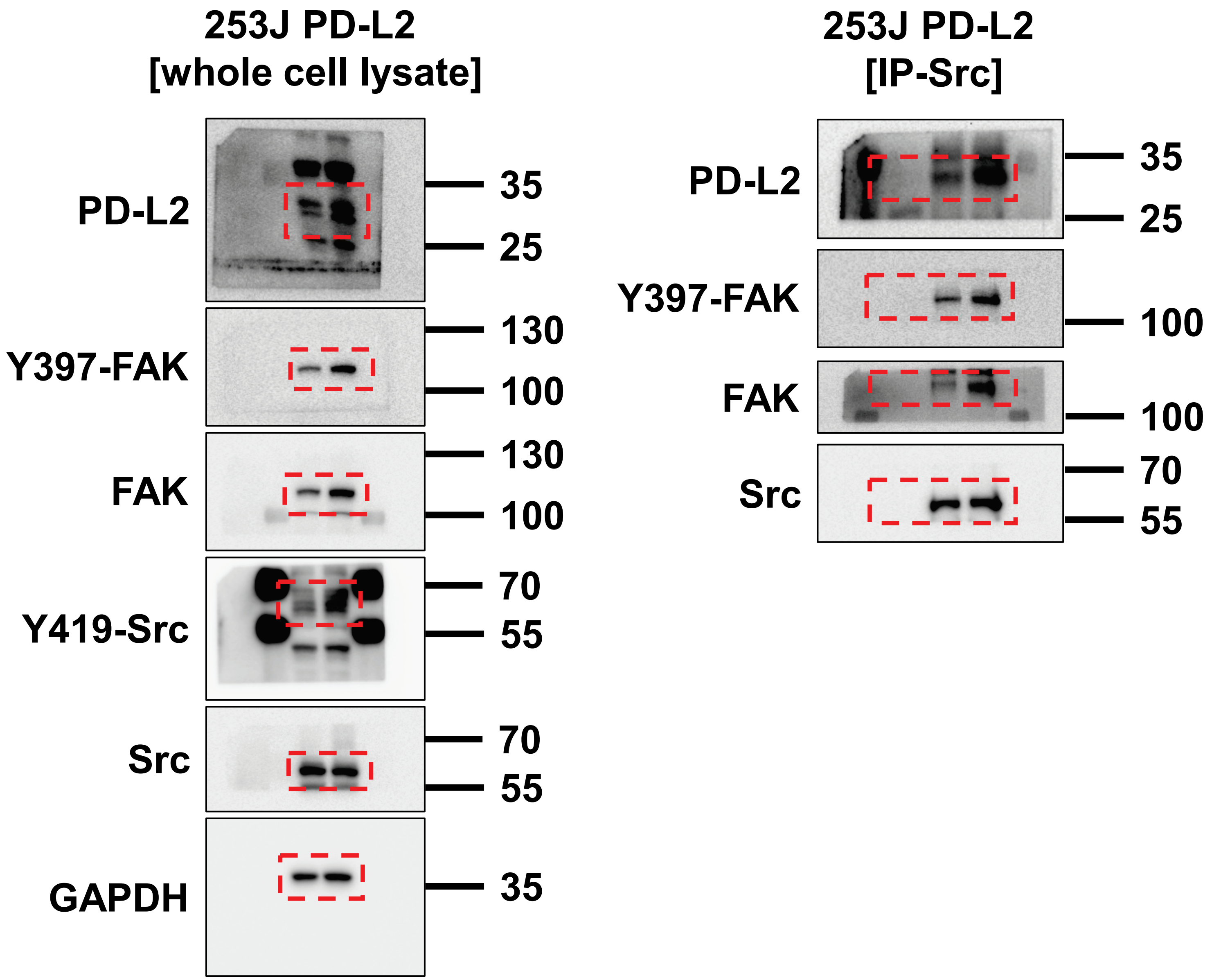
253J PD-L2
[whole cell lysate]
PD-L2
35
25
Y397-FAK
130
100
FAK
130
100
Y419-Src
70
55
Src
70
55
GAPDH
35
253J PD-L2
[IP-Src]
PD-L2
35
25
Y397-FAK
100
FAK
100
Src
70
55

Figure 6F

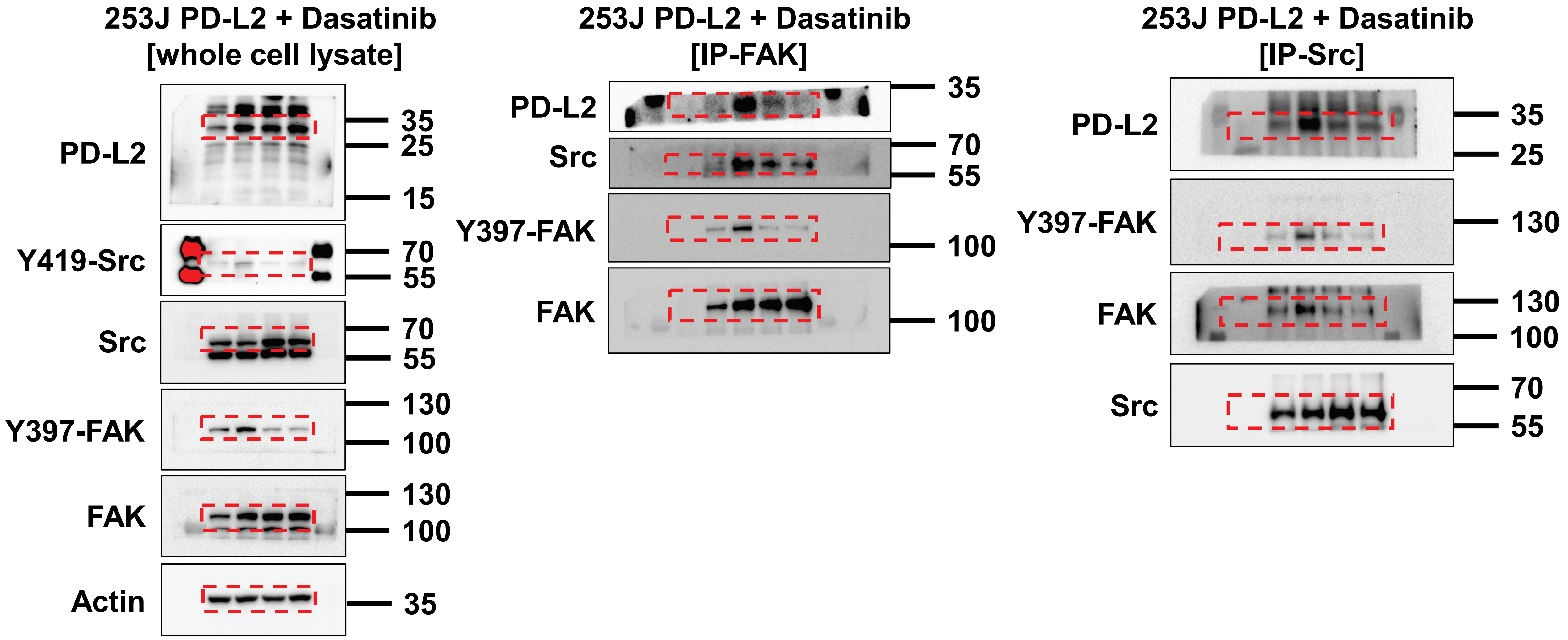

253J PD-L2 + Dasatinib
[whole cell lysate]
PD-L2
35
25
15
Y419-Src
70
55
Src
70
55
Y397-FAK
130
100
FAK
130
100
Actin
35
253J PD-L2 + Dasatinib
[IP-FAK]
PD-L2
35
Src
70
55
Y397-FAK
100
FAK
100
253J PD-L2 + Dasatinib
[IP-Src]
PD-L2
35
25
Y397-FAK
130
FAK
130
100
Src
70
55
